# Speaking valve with integrated biomimetic overpressure release and acoustic warning signal

**DOI:** 10.1038/s41598-024-77595-0

**Published:** 2024-11-04

**Authors:** N. Knorr, P. Auth, S. Kruppert, C. A. Stahl, K. M. Lücking, F. Tauber, T. Speck

**Affiliations:** 1 Plant Biomechanics Group, Botanic Garden, University of Freiburg, Freiburg, Germany; 2 Cluster of Excellence livMatS @ FIT - Freiburg Center for Interactive Materials and Bioinspired Technologies, University of Freiburg, Freiburg, Germany; 3 Division of Medical Physics, Department of Diagnostic and Interventional Radiology, Medical Center, Faculty of Medicine, University of Freiburg, Freiburg, Germany; 4 Institute for Wood Sciences, Biomimetics based on lignocelluloses, University Hamburg, Hamburg, Germany; 5 Medical Center, Faculty of Medicine, University of Freiburg, Freiburg, Germany; 6 Freiburg Materials Research Center (FMF), University of Freiburg, Freiburg, Germany

**Keywords:** Bioinspired, Biomimetic, GLMM, 3D-printing, Speaking valve, Tracheostomy, *Utricularia*, Overpressure release, Quality of life, Biomedical engineering, Plant physiology

## Abstract

Speaking valves enable tracheostomy patients to speak naturally. However, improper use may cause dangerous overpressure, leading to severe complications or even patient’s death. We address this life-threatening issue by creating a biomimetic speaking valve, which incorporates an integrated overpressure valve that automatically opens when reaching critical pressure levels. To enhance safety, we integrated a whistle module to provide an audible alert for medical staff. Fundamental research on the *Utricularia vulgaris* trapdoor inspired our abstracted valve form. Through a comprehensive analysis using generalized linear mixed-effect models, we examined various membrane parameter effects on the function of the biomimetic overpressure valve. This enabled us to adjust the valve’s opening pressure to cater to patient’s unique requirements, thus potentially saving lives by applying a solution from nature.

**Supplementary Information:**

The online version contains supplementary material available at 10.1038/s41598-024-77595-0.

## Introduction

Speaking valves enable tracheotomized patients to vocalize by redirecting exhaled air towards their vocal cords^1–6^. However, applying a speaking valve to a tracheotomy tube with an inadvertently inflated cuff can cause rapid overpressure, leading to pneumothorax (barotrauma), air leak disease, and potentially death^7,8^. As a consequence of at least four reported fatal cases of accidental misuse of speaking valves in Germany alone and an unknown number of unreported cases, the German Federal Institute for Drugs and Medical Devices (BfArM) has prompted manufacturers to enhance the labeling of their devices^9^. However, as we learned from the Critical Incident Reporting System (CIRS) from the University Clinic Freiburg, even experienced intensive care nurses were still inadvertently endangering patients, even after switching to valves labelled in red warning color, as was proposed by the BfArM. Intensive Care Unit (ICU) – patients with reduced levels of consciousness are considered particularly vulnerable to this threat. We concluded that speaking valves should be equipped with a built-in function to protect patients unassisted. We propose that a speaking valve should have an integrated overpressure valve and an acoustic signal generator. Intrathoracic overpressure of more than 40–50 mbar are considered critical in ICU – patients both in the literature^10–13^ and in clinical experience. The valve must therefore open quickly and safely with a sufficiently large opening to prevent these critical pressure levels even when the patient coughs. An audible alarm should alert the caregiver to take measures to remove the danger from the patient^14–16^. However, to avoid disrupting speech, the valve must be airtight below critical pressures.

To meet these medical requirements, we designed a biomimetic speaking valve. This valve features a pressure relief valve inspired by the trapdoor morphology and functioning of the carnivorous bladderwort *Utricularia vulgaris*, which is connected in series with a labial whistle. Our design considers the importance of both functionality and safety, while also drawing inspiration from nature. Since the morphology and functionality of the biological model were not copied but rather its mode of operation was abstracted, our overpressure valve meets the definition of biomimetic development according to VDI 6220 Part 2^17^, see also^18^.

### Functional morphology in *Utricularia vulgaris*

The genus *Utricularia* comprises more than 220 species^19^. The rootless aquatic species obtain nutrients through prey digestion (small nematodes, crustaceans and larvae)^20,21^. Among the different trap types found in the *Utricularia* genus, the *Utricularia* sect. *Utricularia* trap type is the most frequently studied due to the aquatic lifeform of this group^22,23^.

**Fig. 1:**
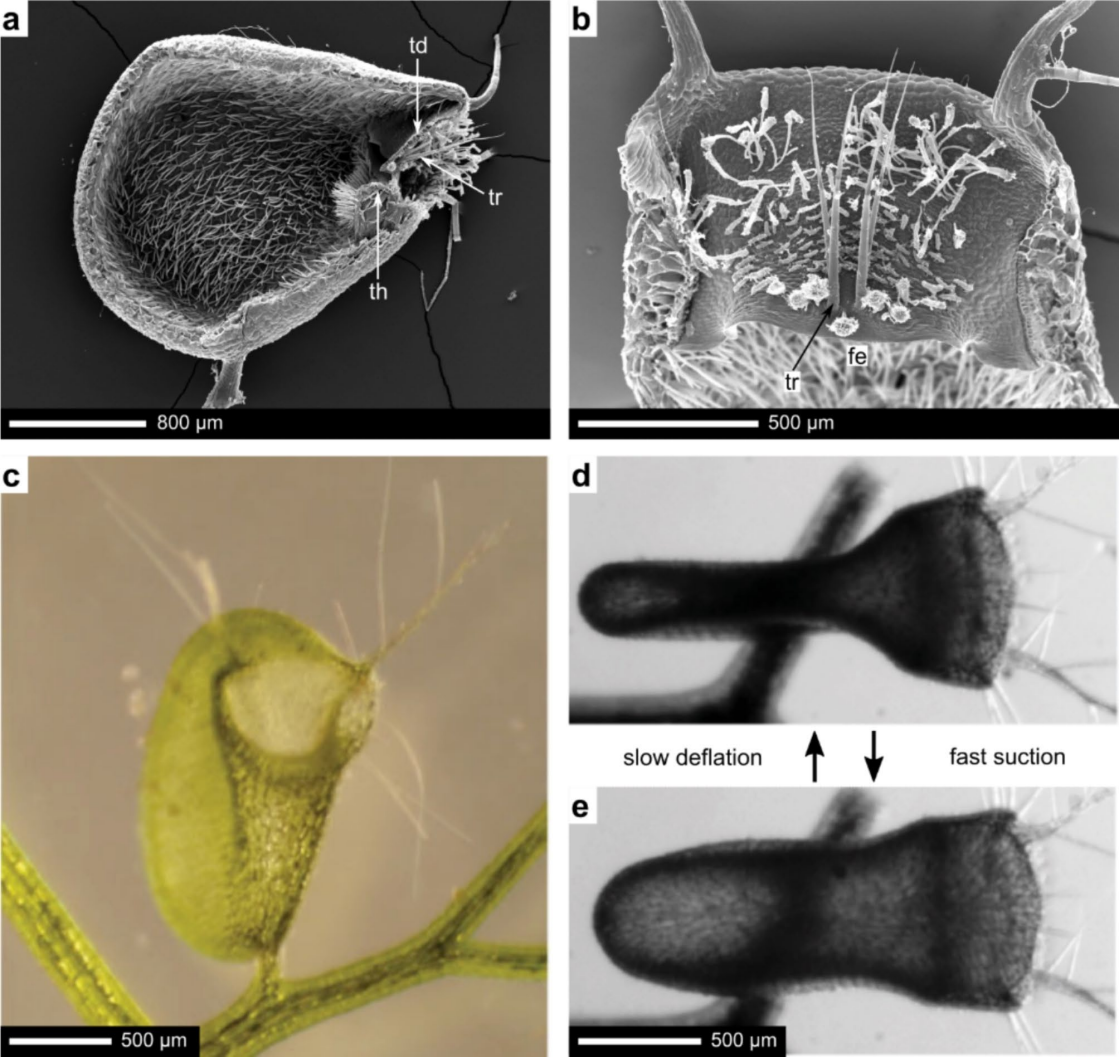
Trap Morphology of *Utricularia*. (**a**) Scanning electron micrograph of a suction trap cross-section with the trapdoor (td), threshold (th) and trigger hairs (tr). (**b**) Scanning electron micrograph of the outer door surface with trigger hairs (tr) located near the free edge (fe). (**c**) Frontal view of a suction trap. (**d,e**) Top view on a suction trap before (**d**) and after (**e**) a trigger event. (**a**) Modified from^24^, (**b–e**) modified with permission of Royal Society Publishing from^25^.

The aquatic suction traps of *Utricularia vulgaris* consist of a hollow, water filled bladder and a connected trapdoor (Fig. 1). The free edge (fe) of the highly sophisticated trapdoor (td) seals the entrance watertight at a threshold (th)^24,25^. To activate the trap, water is pumped out of the bladder, creating an internal underpressure. A tridimensional convex curvature stabilises the trapdoor to withstand this pressure^22^. When small prey contacts one of the trigger hairs (tr), the trapdoor is mechanically destabilized. This triggers an inversion of the trapdoor curvature in a snap-buckling manner^25^. As the lateral trap walls relax, water and prey are quickly and reliably sucked into the trap, equalizing the pressure inside the bladder to ambient water pressure. For a more detailed description of the trapdoor morphology and the opening, please refer to the supplementary document section “Trap system of *Utricularia*”.

## Results

### Abstraction of the key principles of *Utricularia* trap doors for the bioinspired overpressure valve

**Fig. 2:**
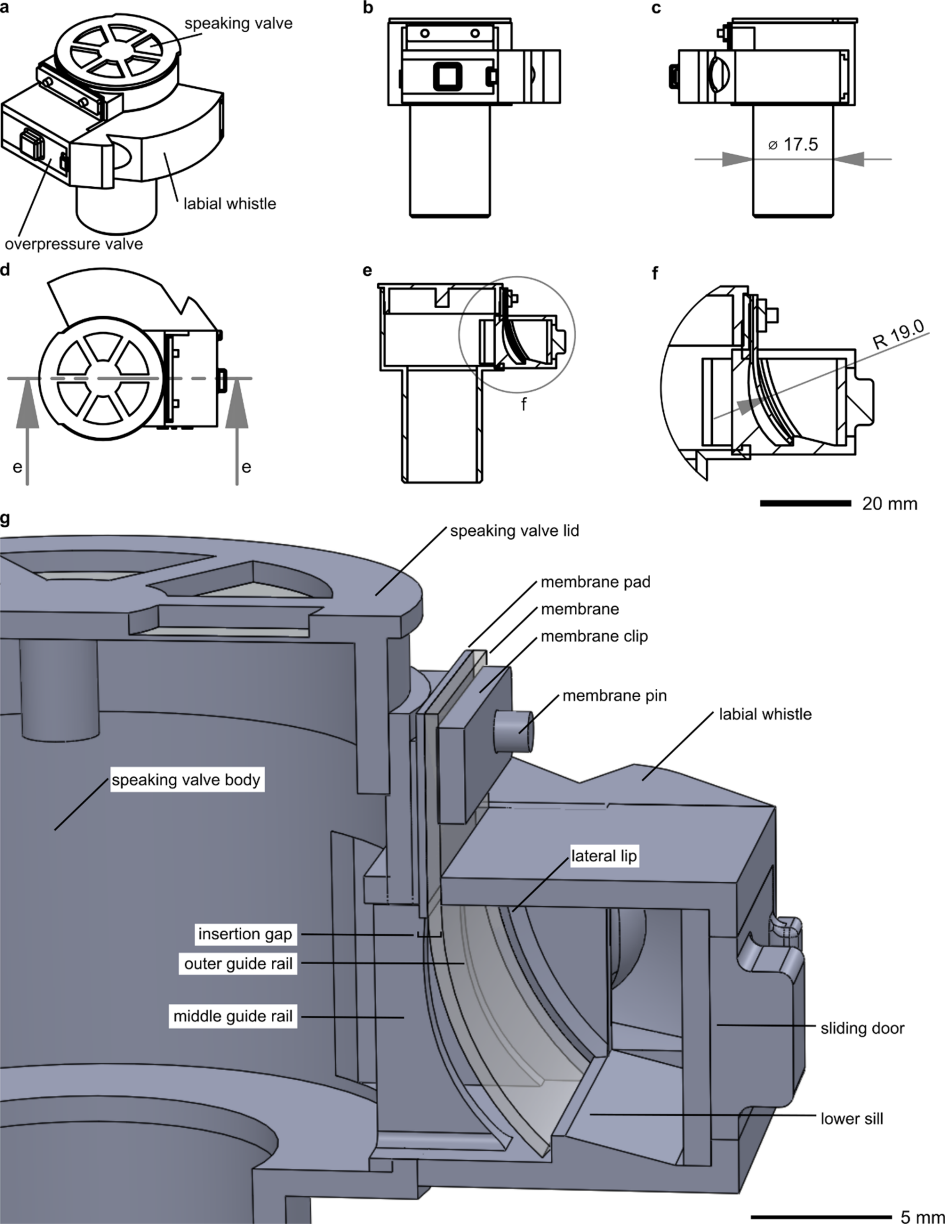
Different views of the biomimetic speaking valve, consisting of a commercially available speaking valve body extended by an overpressure valve and a labial whistle. (**a**) Trimetric view. (**b**) Front view. (**c**) Right side view from **(b)** with the overpressure valve on the left-hand side and front view of the labial whistle. (**d**) Top view with indicated cross section e-e. (**e**) Cross section through the speaking valve body and the lateral overpressure valve with indicated detail view (**f**). (**f**) Detailed view of the cross section through the lateral overpressure valve with a membrane radius of 19 mm. (**g**) Cross section of the speaking valve with overpressure valve and component designation. The biomimetic speaking valve was modeled after a standard commercial speaking valve. The body of the overpressure valve and labial whistle were integrated in the outer wall of the main body of the speaking valve. The membrane (transparent) was inserted from the outside and fixed with a membrane clip on two membrane pins. Using a membrane pad under the membrane, three membrane thicknesses (0.3, 0.5, 0.8 mm) could be tested in the same 3D-printed speaking valve, ensuring an airtight sealing of the insertion gap. The outer and middle guide rails define the radius of the convex membrane curvature. With a rise of pressure, the membrane is pressed against the lateral lip and the lower sill, sealing the valve. In case of an overpressure event the membrane is pushed against the sill, snapping and changing its curvature while opening the overpressure valve. The sliding door is used for better access during the cleaning process after 3D-printing.

The biomimetic overpressure valve was retrofitted to the side of an existing speaking valve design, as seen in Fig. 2. The speaking valve body features a cutout on one side to enable air to enter the overpressure valve and the labial whistle downstream (Fig. 2). This retrofitting guarantees that the primary function of the speaking valve remains unaffected by any potential malfunction of the overpressure valve, as stated in the associated patent application^26^. If overpressure occurs, the membrane of the overpressure valve will open, allowing air to escape through the labial whistle and equalize the pressure. The labial whistle functions as an audible signal to notify caregivers.

The design of the overpressure valve emulates the abstracted functional morphology of the trapdoor. In the biological role model, a three-dimensional convex curvature stabilizes the structure until a critical pressure is reached. However, in a biomimetic membrane creating a three-dimensional curvature turned out to be difficult and expensive as preliminary experiments showed. Our approach with a membrane with convex curvature along only one axis was cost-effective, yielded consistent results, and provided the same functionality, as literature showed^24^. The membrane was inserted from the outside of the overpressure valve through an insertion gap and remained stable until a critical overpressure was reached. Three guide rails, two on either side and one in the middle, directed the membrane into the convex shape (Fig. 2g). The radius of the membrane curvature is easily adjustable by changing the lateral lip radius, described by a semi-circle between the insertion gap and lower sill (Fig. 2f). In *Utricularia*, the velum in the outer threshold zone seals the trapdoor watertight. The lower sill and lateral lips mimic this function by sealing the overpressure valve to prevent leakage currents.

The last abstraction referred to the directional airflow during the pressure equalization after an opening event. In the biological role model, the pressure gradient that is equalized by the trapdoor opening is built up by actively pumping water out of the trap body. The water flows from ambient pressure to low pressure. In the biomimetic overpressure valve, however, the pressure inside the speaking valve body was higher compared to the pressure outside of the valve. Here, the air flows from overpressure inside the valve to ambient pressure outside the valve.

### Valve characterization

**Fig. 3:**
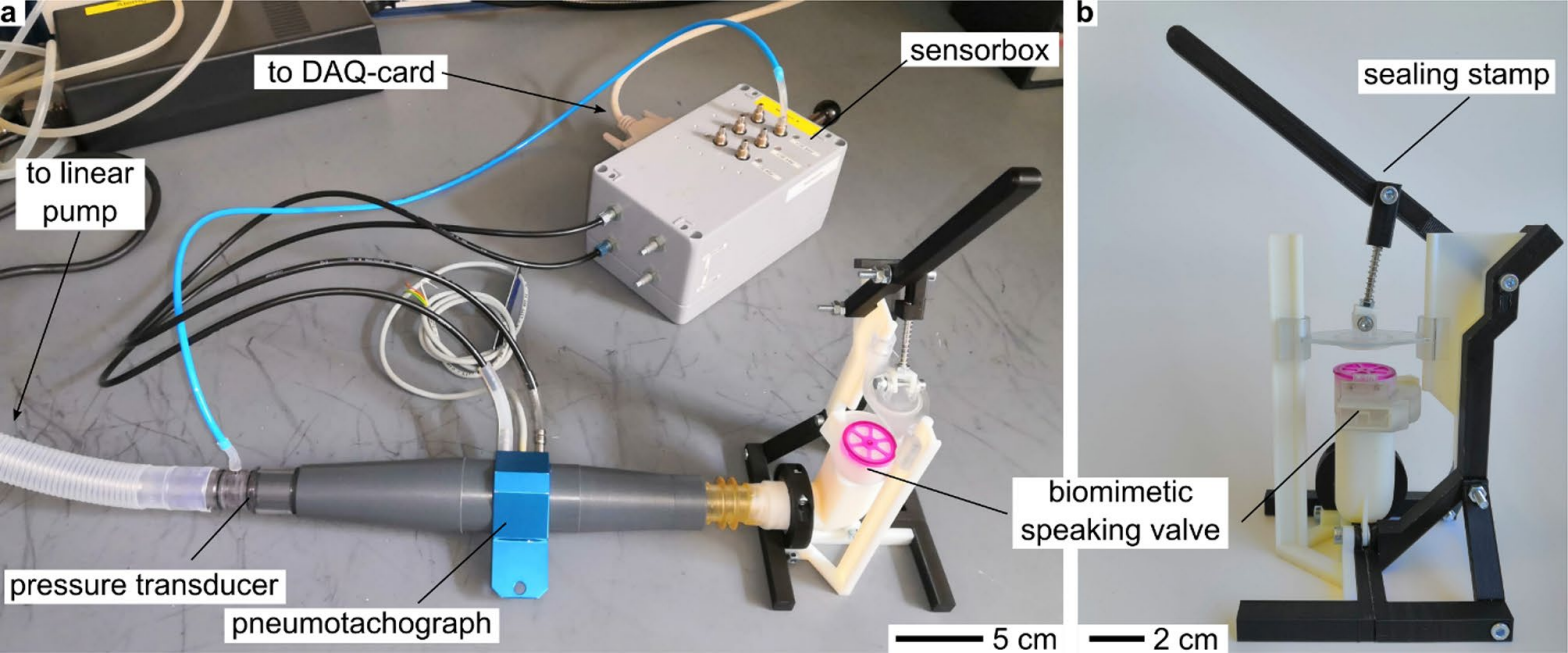
Test bench for testing the biomimetic speaking valve. (**a**) Shows the pressure transducer and pneumotachograph that are connected upstream of the sealing stamp. The sensors are mounted in the sensor box and the connected DAQ-card and linear pump are out of view. (**b**) Shows the sealing stamp with the biomimetic speaking valve.

To investigate the factors that influenced the opening and closing of the overpressure valve and achieve the target pressure range of 40–50 mbar^10–13^, we studied different membrane parameters including thickness, length, and radius of curvature. Ten versions of three biomimetic overpressure valves, each with a different membrane radius of curvature, were designed and tested. The overpressure valve best suited for the intended pressure range was selected for further investigation. We then examined the influence of membrane thickness and length, testing 10 membranes for each parameter combination. The study investigated whether transferring the cell constrictions on the inside of the trapdoor of *Utricularia* to the membrane via laser engraving affects the opening and closing behavior of the pressure relief valve. To conduct the investigation, the valves were connected to an artificial lung (pump with pneumotachograph) (Fig. 3) to simulate inspiration and expiration while measuring the pressure.

### Valve opening and closing

**Fig. 4:**
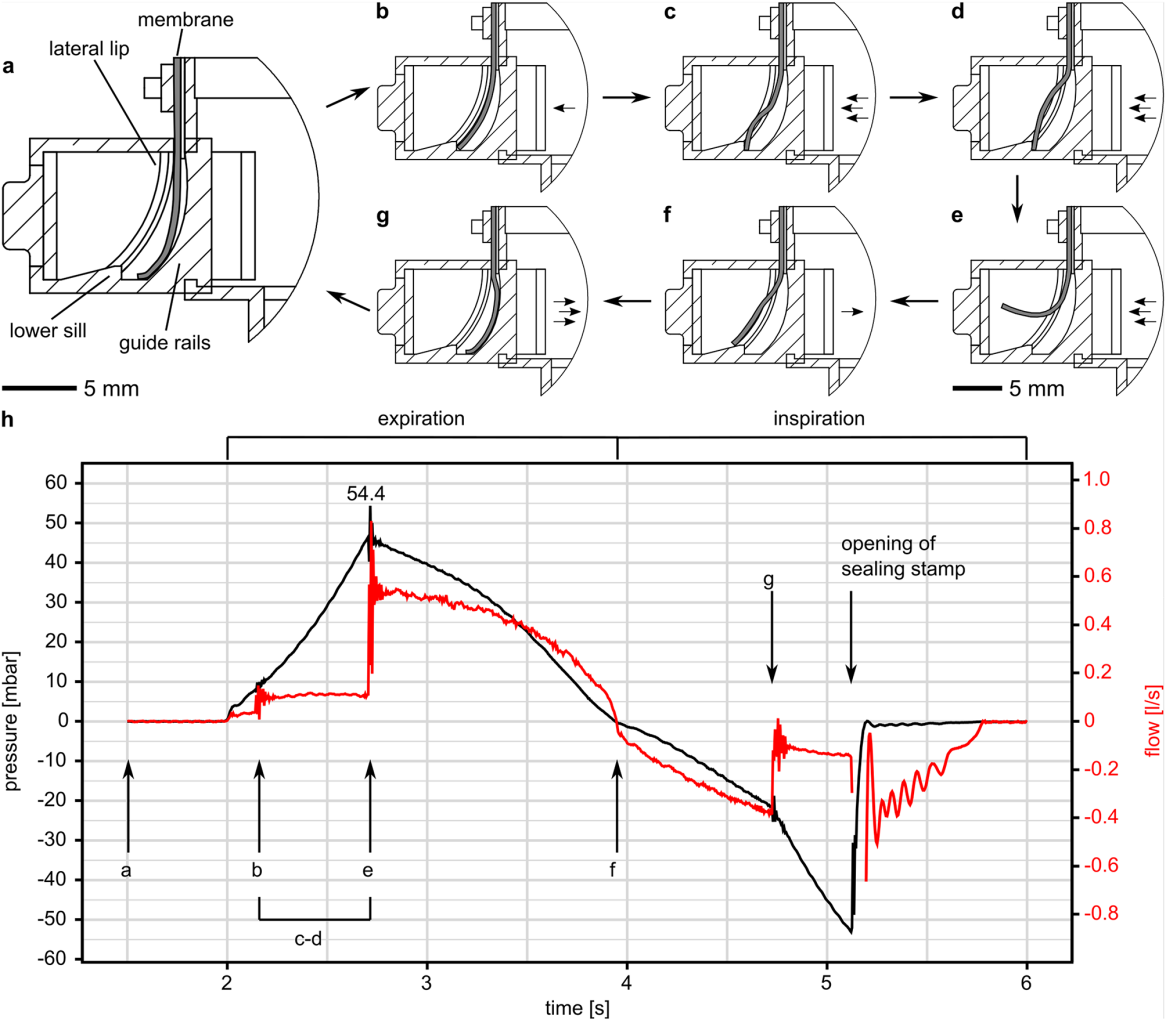
Membrane positions with pressure and flow curves during the opening of the biomimetic speaking valve. The Pictograms (**a–g**) show the membrane position at different points of time during the opening and closing of the overpressure valve. (**h**) Shows the measured pressure and airflow in one respiratory cycle, the membrane positions (**a**–**g**) are labeled. (**a**) Membrane is in the initial starting position. (**b**) The expiration starts, the pressure inside the speaking valve rises, the membrane seals on the lower sill and the lateral lips. (**d**) The pressure rises, the membrane curvature begins to invert near the free edge. (**d**) The convex curvature of the membrane is fully inverted. (**e**) The free edge detaches from the lower sill; the membrane swings open and the pressure equalizes. (**f**) Transition point between expiration and inspiration, the lid of the speaking valve is covered by a sealing stamp. (**g**) The negative pressure inside the speaking valve pulls the membrane over the lateral lips and lower sill. After opening of the sealing stamp the pressure equalizes through the speaking valve lid, the membrane is back in the starting position.

The valve’s opening and closing during and after an overpressure event were characterized using high-speed recordings of the membrane (Movie S1). Figure 4a-g shows pictograms of the overpressure valve’s cross-section for the membrane position during opening and closing. Associated snapshots from the high-speed recording can be seen in Fig. S1. Figure 4h displays a representative pressure and airflow measurement for one respiratory cycle, with states of the membrane motion labeled from a to g. In its initial position, the membrane hung loosely between the guide rails and the lateral lips (Fig. 4a). During simulated expiration, the increasing pressure pushed the membrane against the lateral lips, causing it to deflect into the appropriate radius of curvature (Fig. 4b). As the pressure continued to rise, the membrane bulged and its curvature inverted in a ‘snap buckling’ manner (Fig. 4c and d) (Movie S1). The pressure increased steadily until the membrane opened (Fig. 4e). A sudden peak in airflow was observed, and the pressure inside the speaking valve decreased continuously, limited by the flow rate through the opening of the labial whistle. This produced a constant loud and strident sound until the end of expiration and the start of inspiration (Fig. 4f).

Resetting the membrane required a brief inspiration while sealing the inspiratory opening of the speaking valve; during the experiments, a sealing stamp was utilized (refer to Fig. 3 and Movie S2). As the speaking valve was blocked, the inspiratory airflow passed through the opened overpressure valve. The flow restriction created a negative pressure within the speaking valve, which drew the membrane over the lateral lips and the lower sill back into its initial position (see Fig. 4g). The membrane sealed on the guide rails, resulting in a more rapid drop in pressure and lower flow rate. After opening the sealing stamp, pressure equalized through the speaking valve lid. The overpressure valve was fully reset and ready to open for any further potential overpressure events. Fatigue testing showed, that the opening pressure of the overpressure valve remained consistent over 100 opening events (Fig. S2 in the Supplementary Materials).

### Impact of different membrane parameters on the opening pressure

As theory predicts, we assumed that the opening pressure of the overpressure valve could be controlled by changing the membrane properties — to test this we compared several membrane parameters. Four of these parameters, the membrane radius, thickness, length and engraving, were tested by measuring the opening pressure of a 3D-printed biomimetic speaking valve for every parameter configuration with ten structurally identical membranes. The resulting opening pressures were then examined for significant differences using generalized linear mixed-effects models (GLMM) with the ten structurally identical membranes as a nested random effect and the membrane parameter that was changed between groups as a fixed effect^27–31^.

**Fig. 5:**
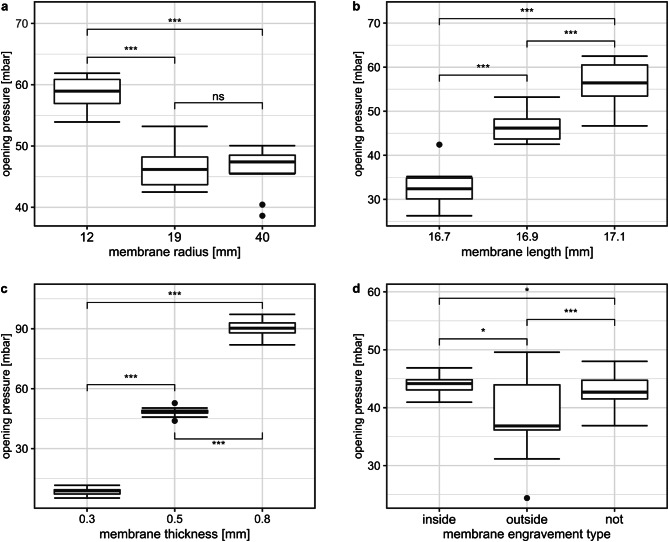
Opening pressures of different membrane parameter configurations. (**a**) Opening pressures of different membrane radii. (**b**) Opening pressures of different membrane lengths tested in an overpressure valve with a 19 mm membrane radius configuration. (**c**) Opening pressures of different membrane thicknesses tested in an overpressure valve with a 19 mm membrane radius configuration. (**d**) Opening pressures of engraved membranes. 19 mm radius corresponds to 16.9 mm length, 0.5 mm thickness and not engraved membrane type. Depicted are the opening pressures of *n* = 10 structurally identical overpressure valves (**a**) and *n* = 10 structurally identical membranes (**b**–**d**) with 25 opening events each per boxplot. Numbers represent p-values from generalized linear mixed-effects models (GLMM), the ten structurally identical membranes were treated as random effect and the membrane parameter as fixed effect. ns: *p* > 0.05, *: *p* ≤ 0.05, **p ≤ 0.01, ***p ≤ 0.005.

The standard membrane configuration was an unengraved 5 mm thick membrane with a radius of 19 mm and a length of 16.9 mm with a mean opening pressure of 46.5 ± 3.4 mbar. A smaller radius of 12 mm led to significantly higher opening pressures (*p* ≤ 0.005, conditional R^2^(10) = 0.97, GLMM) (Fig. 5a). The effect of a higher radius on the opening pressure was not significant in the tested range (*p* = 0.79, conditional R^2^(10) = 0.97, GLMM). Based on these results, we selected the biomimetic speaking valve design with a 19 mm radius for further investigation of different membrane parameters. This valve design achieved reliable opening at the targeted pressure.

Speaking valves with 16.7 mm long membranes showed significantly lower opening pressures compared to 16.9 mm long membranes (*p* ≤ 0.005, conditional R^2^(10) = 0.96, GLMM) (Fig. 5b). The use of 17.1 mm long membranes resulted in significantly higher opening pressures (*p* ≤ 0.005, conditional R^2^(10) = 0.96, GLMM) compared to 16.9 mm membrane length. With a decreased thickness of 0.3 mm, the opening pressure dropped significantly (*p* ≤ 0.005, conditional R^2^(10) = 1.00, GLMM), a higher thickness (0.8 mm) resulted in a significantly higher opening pressure (*p* ≤ 0.005, conditional R^2^(10) = 1.0, GLMM) (Fig. 5c).

As a last parameter, an engraving pattern was abstracted from the cell constrictions in the inner cell layer of the trapdoor and incised using a laser cutter. The pattern on the inside of the membrane (like in *Utricularia*) significantly lowered the opening pressures (*p* = 0.048, conditional R^2^(10) = 0.98, GLMM). Engraving the outside of the membrane also significantly reduced the opening pressure compared to the unengraved membrane (*p* = 0.0039, conditional R^2^(10) = 0.98, GLMM) and compared to the inside engraving (*p* = 0.028, conditional R^2^(10) = 0.98, GLMM) (Fig. 5d). The interquartile range of the engravement on the outside increased threefold to 9.6 mbar compared to the engravement on the inside (3.0 mbar).

## Discussion

The targeted opening pressure for the biomimetic speaking valves was set to 40–50 mbar based on data from medical literature^10–13^. This target was achieved using an unengraved 0.5 mm thick membrane with a radius of 19 mm and a length of 16.9 mm, resulting in a mean opening pressure of 46.5 ± 3.4 mbar. Furthermore, we demonstrated that the opening pressure can be modified by altering the membrane parameters. Our test data confirmed the working hypothesis: stiffer and mechanically more stable membrane parameter configurations result in a higher opening pressure. This is observed with a smaller membrane radius and increased membrane thickness or length. The membrane is fixed at the top and cannot slip forward due to the lower sill. Therefore, the increased membrane length resulted in a slightly tighter bending, having the same effect as a smaller radius. Engraving on the membranes resulted in significant changes in the opening pressure. Regardless of whether the engraving is on the inside or outside of the membrane, some of the resulting opening pressures are lower compared to the unengraved membrane. This can be explained by a reduction of stresses in the bent membrane caused by the engravings. In the case of inside engraving, compressive stresses in the membrane may be reduced, while in the case of outside engraving, tensile stresses may be reduced. However, the stress distribution differs between inside and outside engraving. The cell constrictions on the inside of the curved trapdoor in *U. vulgaris* presumably evolved to make the trapdoor opening more reliable^25^. These constrictions act like a predetermined breaking point, causing the trapdoor to buckle in the center and thus allowing for the largest possible opening of the trapdoor. This reduces the chance of a partial buckling e.g. on only one side of the trapdoor, which would reduce chances of prey capture. The grouping of the opening pressure of the inside engraving shows a similar effect, with a smaller variation compared to the unengraved membrane or the outside engraving. Therefore, the cell constrictions in the *Utricularia* trapdoor and the inside engraving on the membrane of the overpressure valve both enhance the reliability and uniformity of the buckling.

In an overpressure event, the labial whistle produces a constant, loud, and strident sound. This warning signal is activated every time the membrane opens during the experimental phase and has proven to be highly reliable. The distinct sound of the warning is particularly noticeable, as research shows that unique acoustic signals shorten reaction and response time. For instance, pilots are informed which buttons to push to react to distinct acoustic warning signals^32–35^. The sound from the overpressure valve is unique among other audible and visual electronic alarms, making its origin clearly identifiable. Therefore, caregivers can quickly identify it and provide urgent help for the patient.

In the experiments, a sealing stamp was used to reset the membrane in the overpressure valve by sealing the speaking valve lid. In a real-world scenario, the patients can reset the speaking valve themselves by sealing the opening with their palm. This method is both convenient and safe, as it eliminates the need for foreign objects to be inserted into the speaking valve, which could potentially cause contamination or damage of the valve. To ensure ease of implementation for patients, a universal speaking valve with an integrated overpressure valve could be developed, standardizing the implementation process. However, a speaking valve with a customizable opening pressure to suit the specific needs of a patient is preferred. The easiest and cheapest way to adjust the opening pressure is by altering the membrane length. The opening pressure can be conveniently tailored to the patient’s needs by replacing the membrane in the overpressure valve, which can be done by caretaker staff. Additionally, the membranes can be color-coded to differentiate between those with different opening pressures. Altering the thickness yields similar results, albeit with added complexity due to the sealing in the insertion gap. Furthermore, ensuring consistent membrane thickness during assembly posed challenges, requiring meticulous measurement and sorting due to significant variances in thickness observed in the tested membranes.

A direct comparison of the performance between our biomimetic speaking valve and other proposed designs is challenging due to the lack of available characterizations. However, an analysis of the construction of several patented designs^4–6^ highlights distinct advantages of our approach. The structural separation of the inspiration and overpressure valves in our design ensures uninterrupted airflow during inspiration, even in the event of a blockage in the overpressure valve. Additionally, the design is streamlined, with few moving parts, none of which extend beyond the assembly space. This feature prevents external movement from compromising functionality and ensures that the overpressure release mechanism remains unobstructed by external factors. Furthermore, patient posture and with it orientation of the speaking valve does not affect its performance, as the membrane is securely held in place by membrane pins and guide rails, ensuring consistent operation regardless of positioning.

In conclusion, the developed biomimetic speaking valve solves a life-threatening problem with material embodied intelligence. Inspired by the trapdoor mechanism of *Utricularia* trapdoor, the overpressure valve reliably opens in the event of overpressure and releases it to the environment via a whistle module. This not only protects the patient’s lungs from overpressure but also directly alarms medical staff with a loud, distinctive audible signal, which stands out among frequently occurring electronic alarms. This biomimetic development has the potential to save lives with a solution inspired by nature. This study shows the feasibility of our approach. However, as speaking valves are strictly regulated medical devices, considerable development work is still required before they can be used on patients.

## Materials and methods

### Design and additive manufacturing of the biomimetic speaking valve

To ensure a reliable opening of the biomimetic speaking valve within the desired pressure range, three speaking valve designs were developed to test various membrane parameters. The speaking valve demonstrators were modeled using SolidWorks 2019 CAD software (Dassault Systems Deutschland GmbH, Stuttgart, Germany), with dimensions based on a standard speaking valve from Passy Muir Inc (Mitchell South, Irvine USA). The valve body and labial whistle of the overpressure valve were designed to integrate into the wall of the speaking valve body. The membrane in the valve body acted as an ‘artificial trap door’ and sealed the valve body during inspiration. The system was adaptable to different patient conditions and included a changeable membrane for ease of use. The membrane was inserted from the outside of the overpressure valve and secured with a membrane clip on two membrane pins. To test various membrane thicknesses (0.3 mm, 0.5 mm, 0.8 mm) in the same 3D-printed speaking valve, a membrane pad was used under the membrane to ensure a consistent 0.8 mm thickness and seal the insertion gap. The outer and middle guide rails facilitated the membrane’s convex curvature, three curvature radii (12 mm, 19 mm, 40 mm) were tested. The sliding door improved access during the cleaning process after 3D-printing. For a detailed classification of the components see Fig. 2.

The speaking valve demonstrators were printed in VeroClear RGD810 using a PolyJet 3D printer (Objet260 Connex3, Stratasys GmbH, Rheinmünster, Germany). After printing, the demonstrators were post-cured in a UV chamber (BLX-E365, Preqlab Biotechnologie GmbH, Erlangen, Germany) for 10 min at 365 nm. Support material was used in areas with undercuts and overhangs to prevent the printer from printing into empty space. The material was then manually removed using a spatula and a PolyJet Waterjet (OWJ-03EU, Stratasys GmbH, Rheinmünster, Germany). Figure S3 in the Supplementary Materials shows the three cleaning steps.

### Preparation of the membranes

The membranes and membrane pads were cut with a laser cutter (MT 5030W60, Maitech Advanced Machinery, Lonate Pozzolo, Italy) from transparent silicone mats (Greendale Rubber en Kunststoffen, Wissenkerke, Noord Beveland, Netherlands) with thicknesses of 0.3 mm, 0.5 mm and 0.8 mm.

Both smooth membranes and engraved membranes were produced. The engraving pattern was inspired by the concentric cell constrictions on the trapdoor inner surface of *Utricularia vulgaris*. As in the biological model, the engraved line represented an indentation in the surface of the membrane. Unlike in the case of *Utricularia*, it was technically not possible to leave the free edge unengraved. Therefore, the pattern was altered so that the start and end points of the engraved lines were at the edge of the membrane. The designed engraving pattern was overlaid on a scanning electron micrograph of the trapdoor interior in Figure S4.

### Experimental setup

The experimental setup consisted of a linear pump (PS01-48x240F-C, NTI AG LinMot, Spreitenbach, Switzerland) which simulated the function of a lung with an amplitude of 600 ml and a frequency of 15 1/min during the test period (Fig. 3). The parameters were chosen on the basis of literature values and experimental experience from the physicians among the coauthors^12,13^. The pump was controlled in LabView 7.1 (National Instruments, Austin, Texas). Flow through the speaking valve demonstrators was measured with a Fleisch No. 2 pneumotachograph (F + G GmbH, Hechingen, Germany) connected to a differential pressure transducer (PC100 SDSF, Hoffrichter, Schwerin, Germany). Pressure was measured using a piezoresistive pressure transducer (1790, SI-special Instruments, Nördlingen, Germany). The signals were digitized at 200 Hz using an analog-to-digital converter (DAQCard-AI-16E-4, National Instruments, Austin, Texas).

The speaking valves were placed in a sealing stamp setup and connected to the linear pump. Pressure built up during each simulated expiratory phase, triggering the biomimetic overpressure valve and resulting in pressure equalization (see Fig. S1). During the following experiment, the speaking valve lid, which is responsible for allowing unobstructed airflow through the valve, was covered by the sealing stamp. As a result, air entered the valve through the opened overpressure valve, resetting the released membrane back to its initial position. Each membrane was tested for 25 consecutive respiratory cycles. One full breathing cycle with a trigger event and resetting of the membrane can be observed in Movie S1. The sliding door used for cleaning purposes on the backside of the overpressure valve was removed to film the membrane. The high speed videos were recorded in 1600 fps using a Baumer VCXU-13 M (Baumer GmbH, Friedberg, Germany) with HV612M (Space Inc., Sakai Musashino, Tokyo, Japan) and a Veritas Constellation 120E15 (IDT-Integrated Design Tools, Inc., Pasadena, United States). Movie S2 shows opening events and the resetting of the membrane in normal speed with audio.

### Statistical analysis

The opening pressures of the tested membranes were examined for significant differences using generalized linear mixed-effects models (Tables S1–S8). These were created in RStudio using the lme4 package^27,31^. The ten structurally identical membranes per membrane parameter configuration were set as a nested random effect. The membrane parameter that was changed between groups was set as a fixed effect. The opening pressure was the response variable. Post hoc tests with holm p-value adjustment to obtain p-values were conducted using the multcomp package^28^.

## Electronic Supplementary Material

Below is the link to the electronic supplementary material.

Supplementary Material 1

Supplementary Material 2

Supplementary Material 3

Supplementary Material 4

Supplementary Material 5

Supplementary Material 6

Supplementary Material 7

Supplementary movie 1

Supplementary movie 2

## Data Availability

All data supporting the findings of this study are available within the paper and its supplementary information files or are available from the corresponding author upon reasonable request.
