# Supplementary material for: Speaking valve with integrated biomimetic overpressure release and acoustic warning signal
Source: Sci Rep. 2024 Nov 4;14:26655. doi: 10.1038/s41598-024-77595-0 (PMC11535527; doi:10.1038/s41598-024-77595-0)
Supplement: Supplementary file 1 — Supplementary Material 1 [file 41598_2024_77595_MOESM1_ESM.docx]

Supplementary Materials for

**Speaking valve with integrated biomimetic overpressure release and acoustic warning signal**

**Authors:** N. Knorr^1,2,3,4^, P. Auth^1,2^, S. Kruppert^1^, C.A. Stahl^5^, K.M. Lücking^5^, F. Tauber^1,2,^*, T. Speck^1,2,6^

**Affiliations:**

^1^Plant Biomechanics Group, Botanic Garden, University of Freiburg, Germany.

^2^Cluster of Excellence livMatS @ FIT - Freiburg Center for Interactive Materials and Bioinspired Technologies, University of Freiburg, Germany.

^3^Division of Medical Physics, Department of Diagnostic and Interventional Radiology, Medical Center – University of Freiburg, Faculty of Medicine, Freiburg, Germany.

^4^Institute for Wood Sciences, Biomimetics based on lignocelluloses, University Hamburg, Germany.

^5^Medical Center – University of Freiburg, Faculty of Medicine, Freiburg, Germany.

^6^Freiburg Materials Research Center (FMF), University of Freiburg, Germany.

*Corresponding author. Email: falk.tauber@biologie.uni-freiburg.de.

**The PDF file includes:**

Materials and Methods

Supplementary Text

Figs. S1 to S6

Tables S1 to S8

**Other Supplementary Materials for this manuscript include the following:**

Movies S1 to S2

Supplementary Text

Trap system of *Utricularia*

The genus *Utricularia* comprises more than 220 species [36]. This rootless species traps prey (small nematodes, crustaceans and larvae) and digests them to obtain its nutrients [20, 21]. Among the different trap types found in the *Utricularia* genus, the *Utricularia* sect. *Utricularia* trap type is the most frequently studied due to the aquatic lifeform of this group [22, 23].

The trapdoor function of *Utricularia* is highly sophisticated and inspired our design. Oval or spherical modified leaves form a hollow bladder (0.5-6 mm in diameter) which is filled with water and is connected to the plant either directly or via a stalk [22, 36]. The bladder entrance is reinforced by a hinged area connecting the highly sophisticated trapdoor to the upper entrance area [22] (Figure S5 A). The trapdoor is 20-40 μm thick and consists of two cell layers [24] (Figure S5 B). The outer layer is uniform, whereas the inner layer can be divided into different sections (Figure S5 D, E). Concentric cell constrictions in the central region presumably induce a higher flexibility [37, 25]. The free edge of the trapdoor rests on the threshold, which can be divided into three regions: the inner, outer, and middle region. The middle region of the threshold can be further divided into three zones: middle, outer, and inner [38]. There are two bulges within the middle region of the threshold, one between the middle and outer zone and one within the inner zone. The furrow in between takes up the physically longer free edge of the trapdoor, resulting in a convex curvature of the latter [38] (Figure S5 C). Lateral folds improve the form fit of the trapdoor and a velum in the outer zone seals the entry watertight [22, 25].
Bifid glands are presumed to pump water out of the trap, resulting in a pressure difference of -12 to -16 kPa compared to the surrounding water body [39–42]. This causes the flexible lateral walls to deform and store elastic energy in the trap walls. The trapdoor’s convex curvature, extending from the hinge region to the free edge and along the free edge, stabilizes the trapdoor against high pressure [22] (Figure S6). When prey contacts one of the trigger hairs, originating from the outer cell-layer of the trapdoor, the trapdoor opening is initiated: the trigger hair bends and transfers the deformation to the central region, spreading throughout the entire trapdoor in a “snap buckling” manner, similar to the closing mechanism of the Venus Flytrap *Dionea muscipula* [25, 43]. The lateral walls relax due to the resulting pressure equalization, and the prey gets sucked into the trap within 0.5 ms [24], with the lateral fold increasing the displacement area of the trapdoor. The trapdoor closing takes approximately 2.5 ms [25]. Subsequently, quadrifid glands secrete enzymes to digest the immobilized prey [44].

Fig. S1.


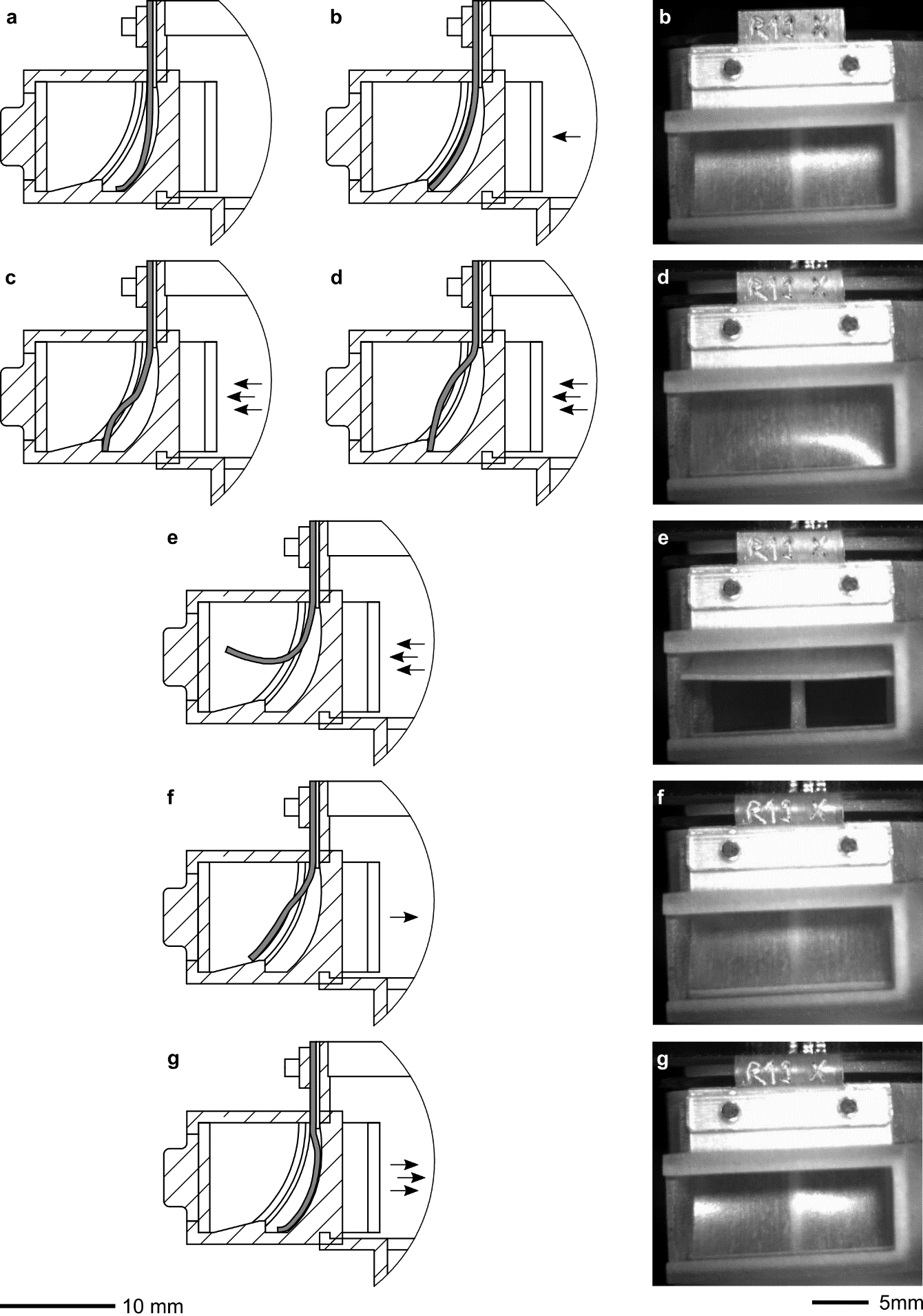


Figure S1: Membrane movement during a trigger event. On the left, the various opening steps are shown as a schematic drawing in cross-section. The arrows indicate the pressure conditions and the direction of flow of the air. On the right, single images from a high-speed recording are shown (Movie S1); the numbers correspond to the same opening step. (a)-(d) show expiration, (e)+(f) show inspiration. (a) Membrane is in the initial position. (b) Membrane is pressed against the lateral lips and the lower sill by slight overpressure, taking on the convex curvature of the lips. (c) Increasing pressure causes the membrane to invert its curvature in a snap-buckling motion, starting from the lower sill. (d) The diaphragm continues to bulge outward until the lower diaphragm edge detaches from the sill and lateral lips. (e) The free edge of the membrane loses contact with the lower sill and flips upwards. (f) The sealing stamp is closed, and the inspiratory airflow runs through the overpressure valve. Slight negative pressure in the speaking valve body presses the membrane against the back of the sill and lateral lips form an airtight seal, thus raising the negative pressure. (g) The membrane is pulled over the lateral lips and lower sill and is pressed against the guide rails. When the sealing stamp is lifted, the pressure equalizes and the membrane changes back to state (a).

Fig. S2.


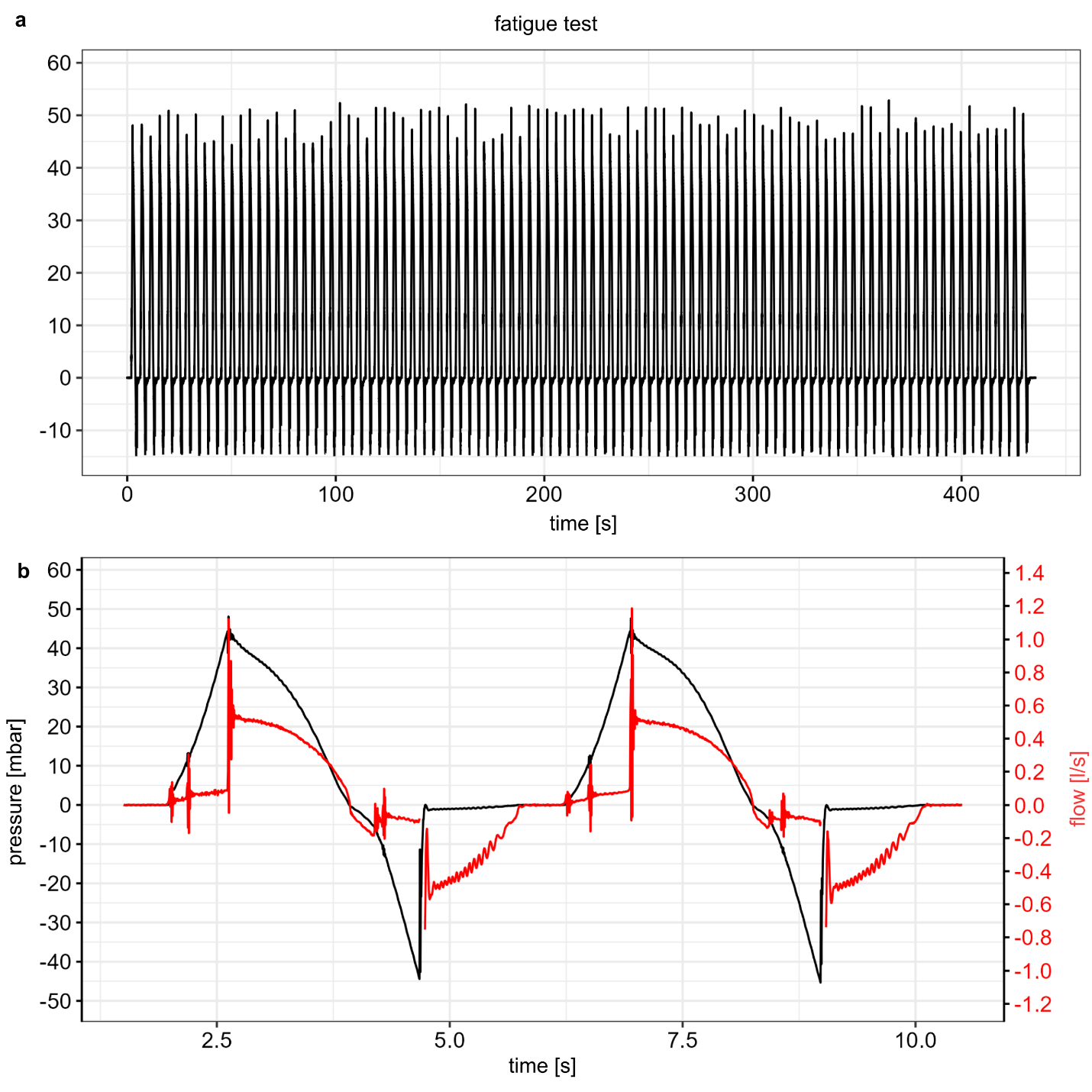


Figure S2: Pressure and flow curves during fatigue testing. (a) shows the the measured pressure during 100 consecutive breathing cycles, (b) shows the measured pressure and flow during the first two breathing cycles from (a).

Fig. S3.


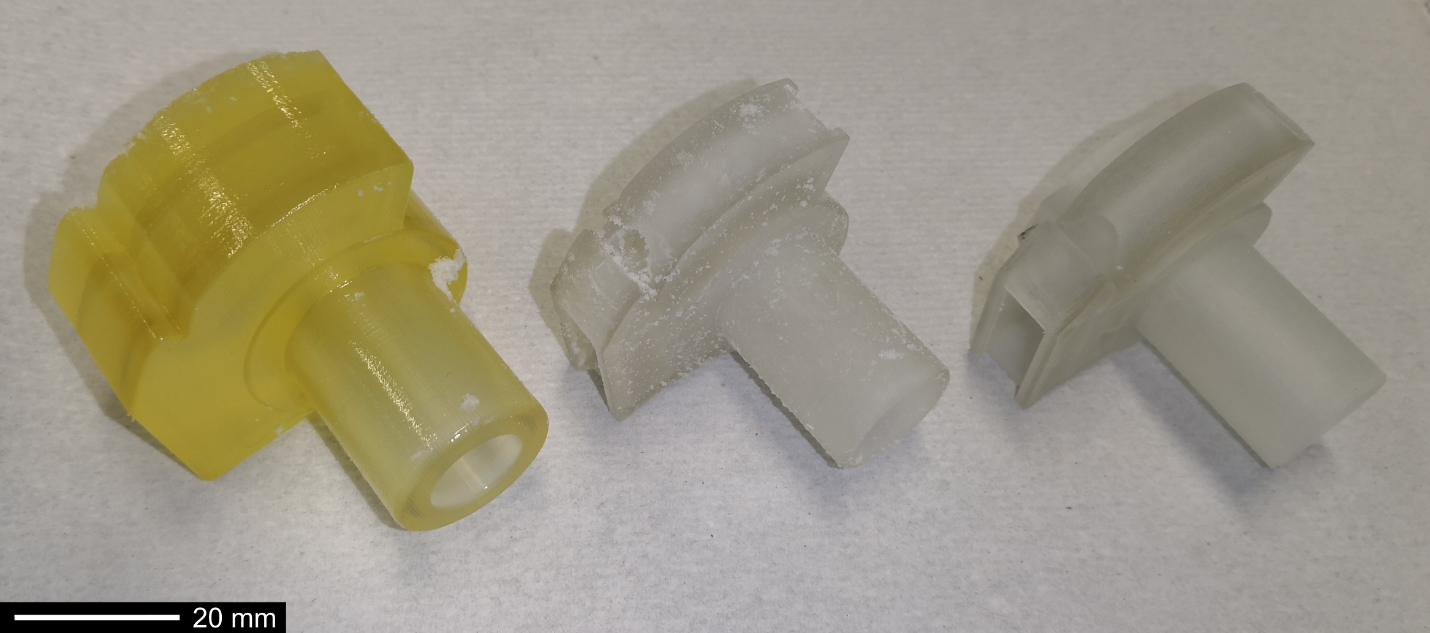


Figure S3: Cleaning steps of the 3D printed biomimetic speaking valves. Shown is a speaking valve directly after printing (left), after manual cleaning with a spatula (center) and after final cleaning with a PolyJet Waterjet (OWJ-03EU, Stratasys GmbH, Rheinmünster, Germany).

Fig. S4.


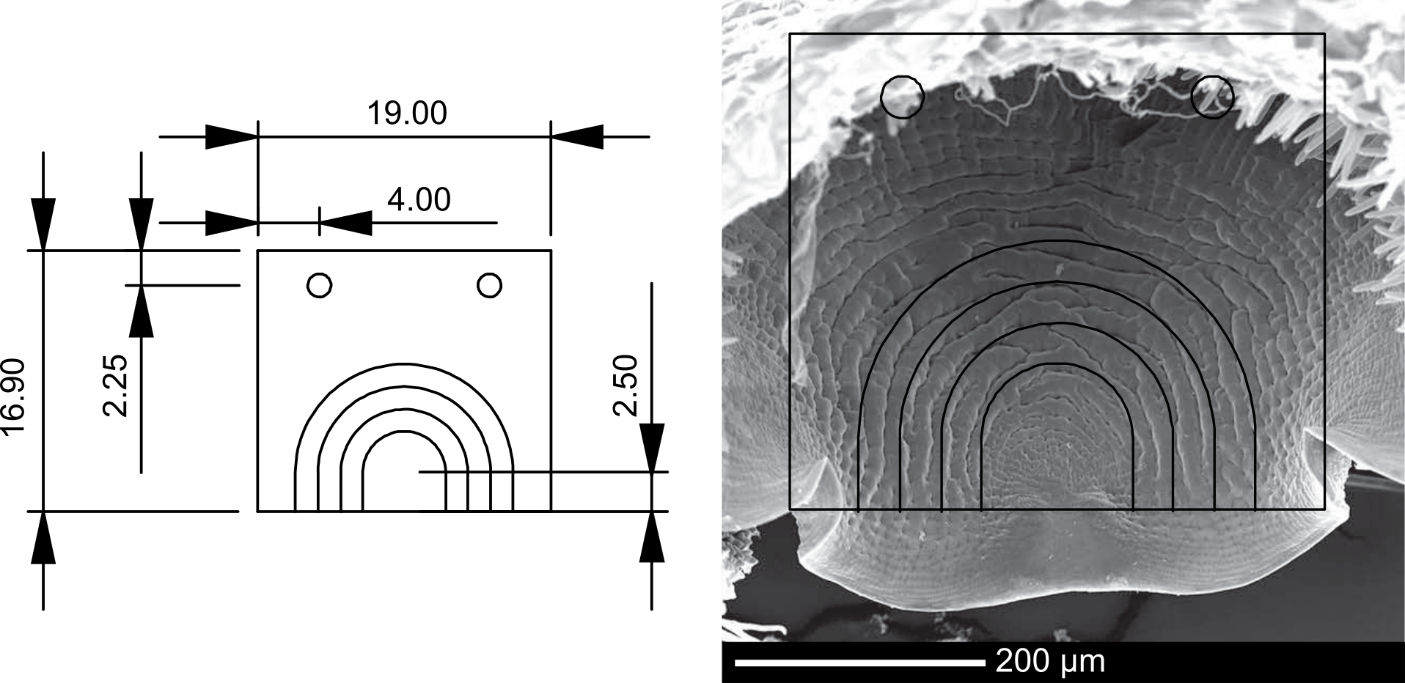


Figure S4: Engraving pattern and cell constrictions on the inside of the trap door of *U. vulgaris*. (Left) Shown are the membrane dimensions in mm and the engraving pattern. (Right) Scanning electron micrograph of the trapdoor interior showing visible cell constrictions of *U. vulgaris* and the engraving pattern as an overlay. Image on the right modified from [24].

Fig. S5.


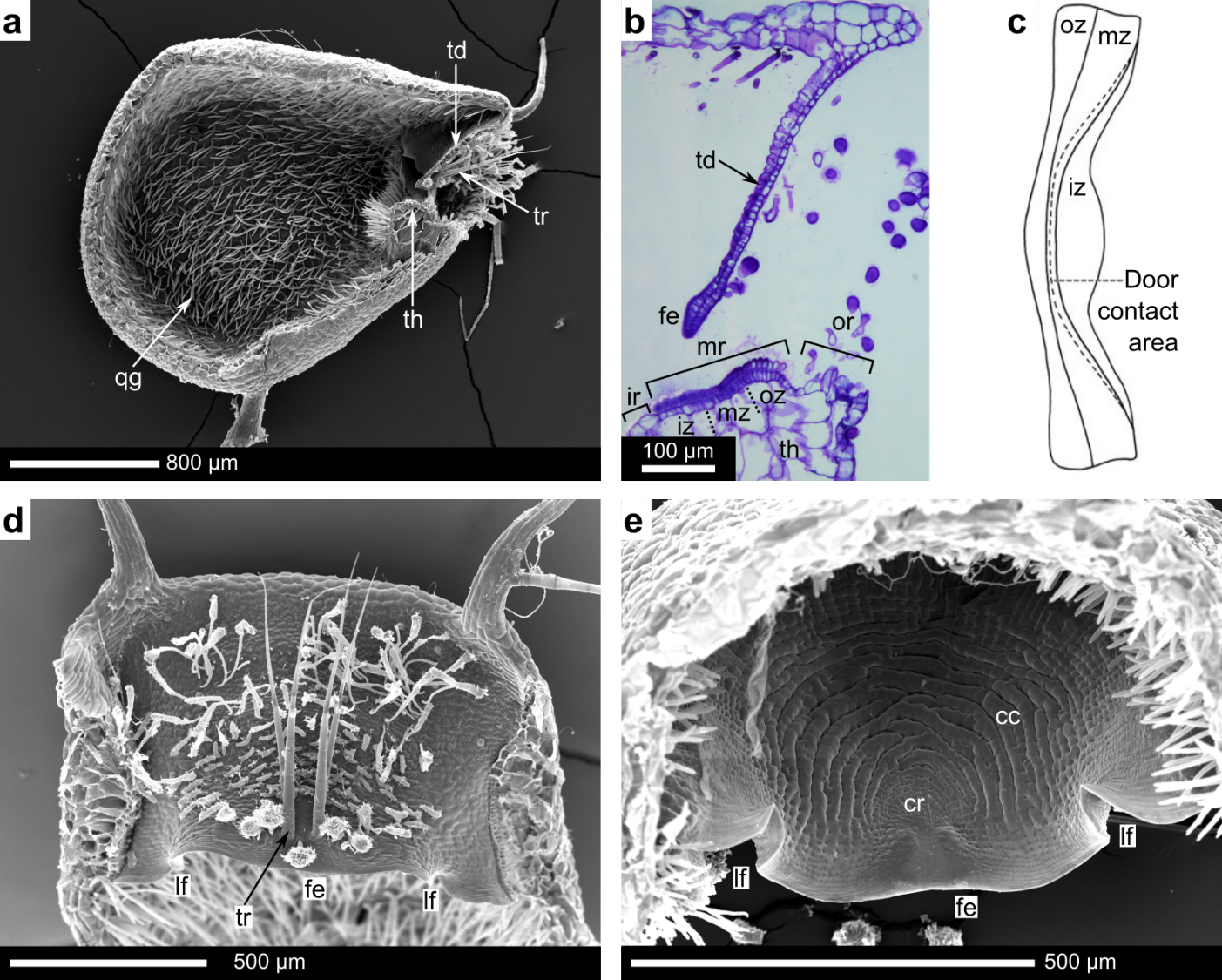


Figure S5: Trap Morphology of *U. vulgaris*. (a) Scanning electron micrograph of a suction trap cross-section with the trapdoor (td), threshold (th), trigger hairs (tr) and quadrifid glands (qg). (b) Light microscopic image of a thin section of the trap entrance with the trapdoor (td), free edge (fe) and the threshold (th), divided in the inner (ir), outer (or) and middle region (mr), which can be further divided into the inner (iz), middle (mz) and outer zone (oz). (c) Schematic view of the threshold with the inner (iz), middle (mz) and outer zone (oz) and the door contact area. (d) Scanning electron micrograph of the outer door surface with trigger hairs (tr), the free edge (fe) and lateral folds (lf) on either side. (e) Scanning electron micrograph of the inner door surface with concentric cell constrictions (cc) around a central region (cr). (a) Modified from [19], (b) modified from [22], (c) modified from [45], (d-e) modified from [20].

Fig. S6.


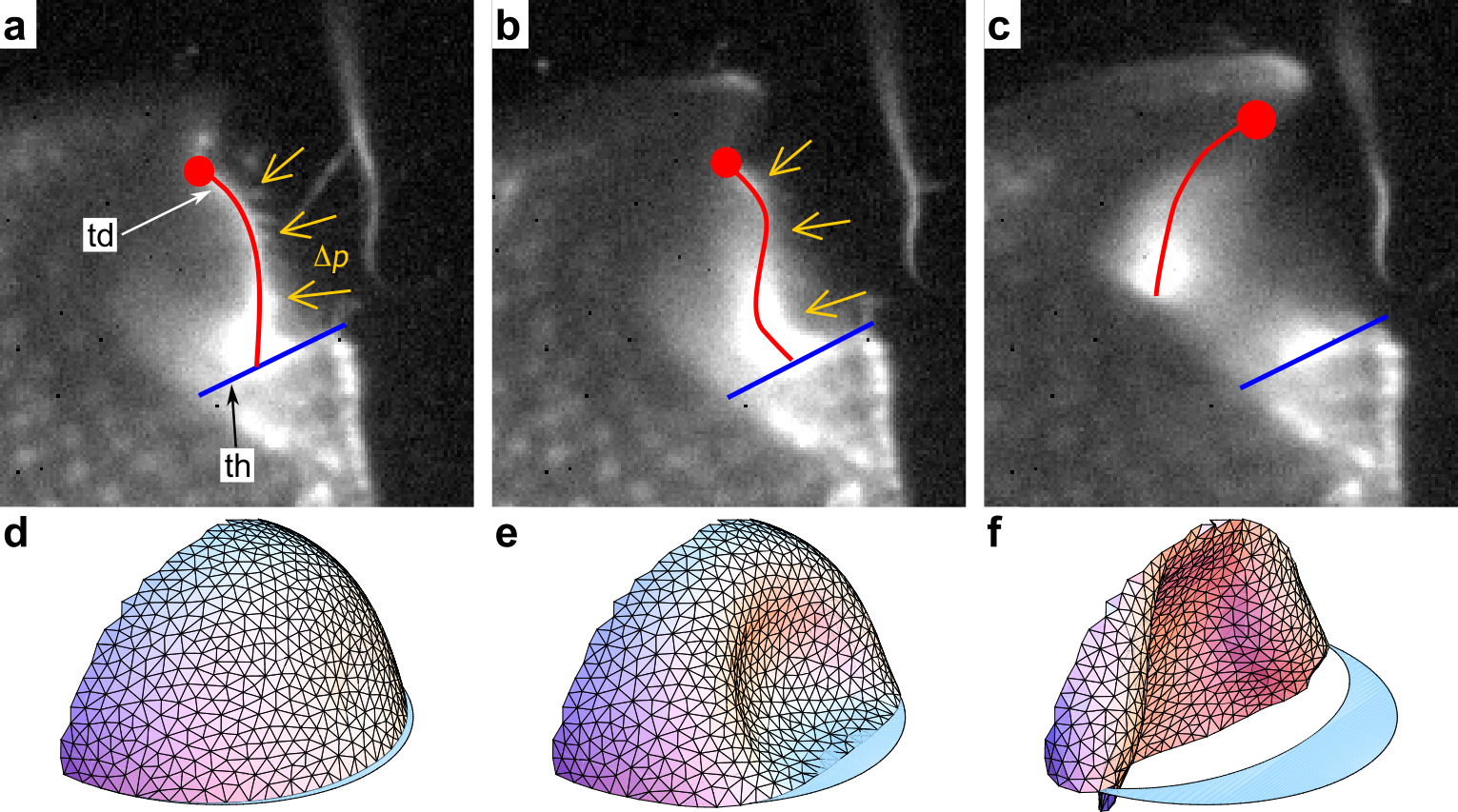


Figure S6: Opening of the trapdoor of U. vulgaris. (a-c) Opening steps of the trapdoor under a light-sheet fluorescence microscope. (d-f) Trapdoor opening in a dynamic simulation. (a) and (d) The trapdoor (td) is closed and forms a tight seal on the threshold (th). (b) and (e) Upon excitation the curvature of the trapdoor is inverted in a “snap buckling” manner, starting at the insertion point of the trigger hair. (c) The door is fully opened. (f) The inversion of the trapdoor curvature is half-way from the start. (a-f) modified from [20].

Table S1.

Table S1: Statistical summary of the generalized linear mixed-effects model (GLMM) to test for statistical differences between the opening pressures of different membrane radii. The ten structurally identical membranes are treated as a nested random effect and the membrane parameter (engravement type) as a fixed effect.

| Term | Est | Type |
| --- | --- | --- |
| (Intercept) | 58.55*** [ 56.95, 60.14] | Fixed Effects |
| fix_19mm | -12.02*** [-14.65, -9.40] | Fixed Effects |
| fix_40mm | -12.46*** [-15.18, -9.74] | Fixed Effects |
| cor_fix_19mm.(Intercept)\|ran | 0.6 | Random Effects |
| cor_fix_40mm.(Intercept)\|ran | 0.08 | Random Effects |
| cor_fix_40mm.fix_19mm\|ran | 0.48 | Random Effects |
| sd_(Intercept)\|ran | 2.56 | Random Effects |
| sd_fix_19mm\|ran | 1.13 | Random Effects |
| sd_fix_40mm\|ran | 2.27 | Random Effects |
| Sigma | 1.11 | Random Effects |
| Model DF | 10 | Overall Model |
| N (Groups) | ran (30) | Overall Model |
| N (Observations) | 750 | Overall Model |
| logLik | -1224.06 | Overall Model |
| AIC | 2468.13 | Overall Model |
| BIC | 2514.33 | Overall Model |
| Marginal R2 | 0.75 | Overall Model |
| Marginal F2 | 2.93 | Overall Model |
| Conditional R2 | 0.97 | Overall Model |
| Conditional F2 | 35.07 | Overall Model |
| fix (Fixed + Random) | 2.93/0.00, p < .001 | Effect Sizes |
| fix (Random) | 0.00/0.00, p = .951 | Effect Sizes |

Table S2.

Table S2: Statistical results of the generalized linear mixed-effects model (GLMM) to test for statistical differences between the opening pressures of different membrane radii. The ten structurally identical membranes are treated as a nested random effect and the membrane parameter (membrane radius) as a fixed effect.

| Simultaneous Tests for General Linear Hypotheses | | | | | |
| --- | --- | --- | --- | --- | --- |
| Multiple Comparisons of Means: Tukey Contrasts | | | | | |
| Fit: lmer(formula = res ~ fix + (fix \| ran), data = membrane radius, REML = F) | | | | | |
| Linear Hypotheses: | | | | | |
| Membrane radius | Estimate | Std. Error | z value | Pr(>\|z\|) |  |
| 19mm - 12mm == 0 | -12.0240 | 1.3399 | -8.974 | <2e-16 | *** |
| 40mm - 12mm == 0 | -12.4558 | 1.3875 | -8.977 | <2e-16 | *** |
| 40mm - 19mm == 0 | -0.4318 | 1.5491 | -0.279 | 0.78 |  |
| Signif. codes: 0 ‘***’ 0.001 ‘**’ 0.01 ‘*’ 0.05 ‘.’ 0.1 ‘ ’ 1 | | | | | |
| (Adjusted p values reported -- holm method) | | | | | |

Table S3.

Table S3: Statistical summary of the generalized linear mixed-effects model (GLMM) to test for statistical differences between the opening pressures of different membrane lengths. The ten structurally identical membranes are treated as a nested random effect and the membrane parameter (membrane length) as a fixed effect.

|  | Est | Type |
| --- | --- | --- |
| (Intercept) | 32.68*** [30.02, 35.35] | Fixed Effects |
| fix_16.9mm | 13.84*** [10.45, 17.23] | Fixed Effects |
| fix_17.1mm | 23.57*** [19.52, 27.63] | Fixed Effects |
| cor_fix_16.9mm.(Intercept)\|ran | -0.63 | Random Effects |
| cor_fix_17.1mm.(Intercept)\|ran | 0.2 | Random Effects |
| cor_fix_17.1mm.fix_16.9mm\|ran | -0.02 | Random Effects |
| sd_(Intercept)\|ran | 4.28 | Random Effects |
| sd_fix_l16.9mm\|ran | 2.33 | Random Effects |
| sd_fix_l17.1mm\|ran | 1.71 | Random Effects |
| Sigma | 2.09 | Random Effects |
| Model DF | 10 | Overall Model |
| N (Groups) | ran (30) | Overall Model |
| N (Observations) | 744 | Overall Model |
| logLik | -1672.39 | Overall Model |
| AIC | 3364.77 | Overall Model |
| BIC | 3410.89 | Overall Model |
| Marginal R2 | 0.81 | Overall Model |
| Marginal F2 | 4.18 | Overall Model |
| Conditional R2 | 0.96 | Overall Model |
| Conditional F2 | 25.43 | Overall Model |
| fix (Fixed + Random) | 4.18/0.00, p < .001 | Effect Sizes |
| fix (Random) | 0.00/0.00, p = .922 | Effect Sizes |

Table S4.

Table S4: Statistical results of the generalized linear mixed-effects model (GLMM) to test for statistical differences between the opening pressures of different membrane lengths. The ten structurally identical membranes are treated as a nested random effect and the membrane parameter (membrane length) as a fixed effect.

| Simultaneous Tests for General Linear Hypotheses | | | | | |
| --- | --- | --- | --- | --- | --- |
| Multiple Comparisons of Means: Tukey Contrasts | | | | | |
| Fit: lmer(formula = res ~ fix + (fix \| ran), data = membrane length, REML = F) | | | | | |
| Linear Hypotheses: | | | | | |
| Membrane length | Estimate | Std. Error | z value | Pr(>\|z\|) |  |
| 16.9mm - 16.7mm == 0 | 13.839 | 1.728 | 8.009 | 2.22e-15 | *** |
| 17.1mm - 16.7mm == 0 | 23.573 | 2.070 | 11.389 | < 2e-16 | *** |
| 17.1mm - 16.9mm == 0 | 9.733 | 1.889 | 5.153 | 2.56e-07 | *** |
| Signif. codes: 0 ‘***’ 0.001 ‘**’ 0.01 ‘*’ 0.05 ‘.’ 0.1 ‘ ’ 1 | | | | | |
| (Adjusted p values reported -- holm method) | | | | | |

Table S5.

Table S5: Statistical summary of the generalized linear mixed-effects model (GLMM) to test for statistical differences between the opening pressures of different membrane thicknesses. The ten structurally identical membranes are treated as a nested random effect and the membrane parameter (thickness) as a fixed effect.

| Term | Est | Type |
| --- | --- | --- |
| (Intercept) | 8.47*** [ 7.30, 9.65] | Fixed Effects |
| fix_0.5mm | 38.05*** [35.65, 40.44] | Fixed Effects |
| fix_0.8mm | 81.66*** [78.75, 84.57] | Fixed Effects |
| cor_fix_0.5mm.(Intercept)\|ran | 0.62 | Random Effects |
| cor_fix_0.8mm.(Intercept)\|ran | 0.71 | Random Effects |
| cor_fix_0.8mm.fix_0.5mm\|ran | 0.34 | Random Effects |
| sd_(Intercept)\|ran | 1.88 | Random Effects |
| sd_fix_0.5mm\|ran | 1.85 | Random Effects |
| sd_fix_0.8mm\|ran | 2.74 | Random Effects |
| Sigma | 1.11 | Random Effects |
| Model DF | 10 | Overall Model |
| N (Groups) | ran (30) | Overall Model |
| N (Observations) | 749 | Overall Model |
| logLik | -1221.55 | Overall Model |
| AIC | 2463.1 | Overall Model |
| BIC | 2509.29 | Overall Model |
| Marginal R2 | 0.99 | Overall Model |
| Marginal F2 | 90.2 | Overall Model |
| Conditional R2 | 1 | Overall Model |
| Conditional F2 | 905.87 | Overall Model |
| fix (Fixed + Random) | 90.20/0.00, p < .001 | Effect Sizes |
| fix (Random) | 0.00/0.00, p = .300 | Effect Sizes |

Table S6.

Table S6: Statistical results of the generalized linear mixed-effects model (GLMM) to test for statistical differences between the opening pressures of different membrane thicknesses. The ten structurally identical membranes are treated as a nested random effect and the membrane parameter (thickness) as a fixed effect.

| Simultaneous Tests for General Linear Hypotheses | | | | | |
| --- | --- | --- | --- | --- | --- |
| Multiple Comparisons of Means: Tukey Contrasts | | | | | |
| Fit: lmer(formula = res ~ fix + (fix \| ran), data = membrane thickness, REML = F) | | | | | |
| Linear Hypotheses: | | | | | |
| Membrane thickness | Estimate | Std. Error | z value | Pr(>\|z\|) |  |
| 0.5mm - 0.3mm == 0 | 38.047 | 1.222 | 31.13 | <2e-16 | *** |
| 0.8mm - 0.3mm == 0 | 81.656 | 1.485 | 54.99 | <2e-16 | *** |
| 0.8mm - 0.5mm == 0 | 43.610 | 1.726 | 25.26 | <2e-16 | *** |
| Signif. codes: 0 ‘***’ 0.001 ‘**’ 0.01 ‘*’ 0.05 ‘.’ 0.1 ‘ ’ 1 | | | | | |
| (Adjusted p values reported -- holm method) | | | | | |

Table S7.

Table S7: Statistical summary of the generalized linear mixed-effects model (GLMM) to test for statistical differences between the opening pressures of different engravement types. The ten structurally identical membranes are treated as a nested random effect and the membrane parameter (engravement type) as a fixed effect.

|  | Est | Type |
| --- | --- | --- |
| (Intercept) | 44.16*** [43.11, 45.22] | Fixed Effects |
| fix_not | 2.36 [0.02, 4.70] | Fixed Effects |
| fix_outside | -5.80* [-10.42, -1.17] | Fixed Effects |
| cor_fix_not.(Intercept)\|ran | 0.94 | Random Effects |
| cor_fix_outside.(Intercept)\|ran | 0.89 | Random Effects |
| cor_fix_outside.fix_not\|ran | 0.67 | Random Effects |
| sd_(Intercept)\|ran | 1.69 | Random Effects |
| sd_fix_not\|ran | 1.73 | Random Effects |
| sd_fix_outside\|ran | 5.72 | Random Effects |
| sigma | 0.79 | Random Effects |
| Model DF | 10 | Overall Model |
| N (Groups) | ran (30) | Overall Model |
| N (Observations) | 750 | Overall Model |
| logLik | -975.56 | Overall Model |
| AIC | 1971.11 | Overall Model |
| BIC | 2017.31 | Overall Model |
| Marginal R2 | 0.34 | Overall Model |
| Marginal F2 | 0.51 | Overall Model |
| Conditional R2 | 0.98 | Overall Model |
| Conditional F2 | 55.26 | Overall Model |
| fix (Fixed + Random) | 0.51/0.00, p < .001 | Effect Sizes |
| fix (Random) | 0.00/0.00, p = .002 | Effect Sizes |

Table S8.

Table 8: Statistical results of the generalized linear mixed-effects model (GLMM) to test for statistical differences between the opening pressures of different engravement types. The ten structurally identical membranes are treated as a nested random effect and the membrane parameter (engravement type) as a fixed effect.

| Simultaneous Tests for General Linear Hypotheses | | | | | |
| --- | --- | --- | --- | --- | --- |
| Multiple Comparisons of Means: Tukey Contrasts | | | | | |
| Fit: lmer(formula = res ~ fix + (fix \| ran), data = engravement type, REML = F) | | | | | |
| Linear Hypotheses: | | | | | |
| Membrane engravement | Estimate | Std. Error | z value | Pr(>\|z\|) |  |
| not - inside == 0 | 2.357 | 1.193 | 1.975 | 0.04823 | * |
| outside - inside == 0 | -5.796 | 2.360 | -2.456 | 0.02812 | * |
| outside - not == 0 | -8.153 | 2.533 | -3.219 | 0.00387 | ** |
| Signif. codes: 0 ‘***’ 0.001 ‘**’ 0.01 ‘*’ 0.05 ‘.’ 0.1 ‘ ’ 1 | | | | | |
| (Adjusted p values reported -- holm method) | | | | | |

Movie S1.


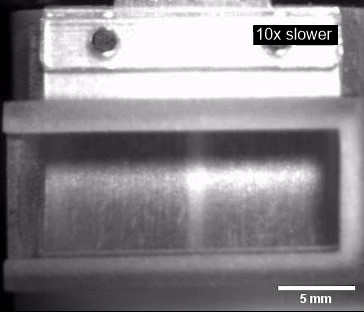


Movie S1: Slow motion video of the opening and closing of the overpressure valve. Shown is the front view of the membrane with the membrane clip on the top securing the membrane on the membrane pins. The different steps of the membrane opening and closing are also depicted in Figure S1. Recorded in 1600 fps using a Baumer VCXU-13M (Baumer GmbH, Friedberg, Germany) with HV612M (Space Inc., Sakai Musashino, Tokyo, Japan) and a Veritas Constellation 120E15 (IDT-Integrated Design Tools, Inc., Pasadena, United States).

Movie S2.


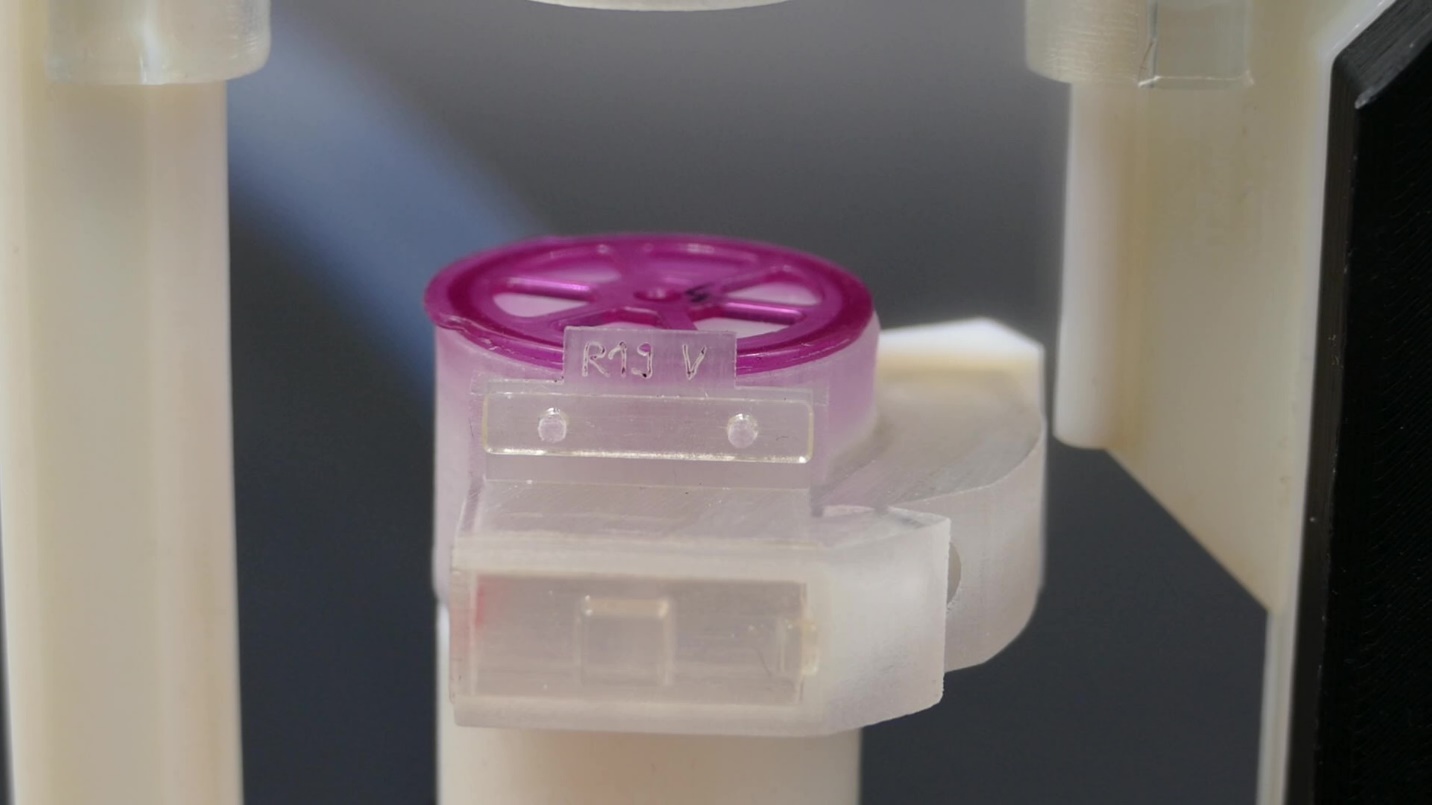


Movie S2: Video with audio of the working biomimetic speaking valve. Shown is the front view of the speaking valve mounted in the sealing stamp. The first exhalation caused an opening of the membrane. The following inhalation resets the overpressure valve due to the sealed speaking valve lid. The second exhalation caused another opening event, the following inhalation passes unobstructed through the speaking valve lid, and the membrane stays open for the third exhalation, which can be heard in the audio. Recorded using a Panasonic DMC-FZ1000 (Panasonic Corporation, Kadoma, Japan).

References

12. G. Grasselli *et al.,* Mechanical ventilation parameters in critically ill COVID-19 patients: a scoping review. *Critical care (London, England)*. **25**, 115 (2021), doi:10.1186/s13054-021-03536-2.

13. L. Fornataro-Clerici, D. J. Zajac, Aerodynamic characteristics of tracheostomy speaking valves. *Journal of speech and hearing research*. **36**, 529–532 (1993), doi:10.1044/jshr.3603.529.

19. P. Taylor, *The genus Utricularia*, *A taxonomic monograph* (Royal Botanic Gardens Kew, London, ed. 1, 1989).

20. E. Brumpt, Capture des larves de Culicides par les plantes du genre *Utricularia*. *Annales de Parasitologie Humaine et Comparée*. **3**, 403–411 (1925).

21. H. N. Moseley, A Carnivorous Plant Preying on Vertebrata. *Nature*. **30**, 81 (1884), doi:10.1038/030081a0.

22. S. Poppinga, U. Bauer, T. Speck, A. G. Volkov, in *Carnivorous Plants, Physiology, Ecology, and Evolution*, A. M. Ellison, L. Adamec, Eds. (Oxford University Press, 2018), vol. **1,** pp. 180–193.

23. A. S. Westermeier *et al.,* Trap diversity and character evolution in carnivorous bladderworts (*Utricularia*, Lentibulariaceae). *Sci Rep*. **7**, 12052 (2017), doi:10.1038/s41598-017-12324-4.

24. S. Poppinga, C. Weisskopf, A. S. Westermeier, T. Masselter, T. Speck, Fastest predators in the plant kingdom: functional morphology and biomechanics of suction traps found in the largest genus of carnivorous plants. *AoB PLANTS*. **8**, plv140 (2015), doi:10.1093/aobpla/plv140.

25. O. Vincent *et al.,* Ultra-fast underwater suction traps. *Proceedings. Biological sciences*. **278**, 2909–2914 (2011), doi:10.1098/rspb.2010.2292.

28. H. Schielzeth, S. Nakagawa, Nested by design: model fitting and interpretation in a mixed model era. *Methods in Ecology and Evolution*. **4**, 14–24 (2013), doi:10.1111/j.2041-210x.2012.00251.x.

29. A. F. Siegel, in *Practical Business Statistics* (Elsevier, 2016), pp. 355–418.

36. R. W. Jobson, P. C. Baleeiro, C. Guisande, in *Carnivorous Plants, Physiology, Ecology, and Evolution*, A. M. Ellison, L. Adamec, Eds. (Oxford University Press, 2018), vol. **1,** pp. 89–104.

37. F. E. Lloyd, *The Carnivorous Plants.* (Chronica Botanica, Waltham, MA, USA., 1942).

38. F. E. Lloyd, Is the door of *Utricularia* an irritable mechanism? *Canadian Journal of Research*. **7**, 386–425 (1932), doi:10.1139/cjr32-091.

39. A. Sasago, T. Sibaoka, Water extrusion in the Water extrusion in the trap bladders of *Utricularia vulgaris* I. A possible pathway of water across the bladder wall. *The Botanical Magazine, Tokyo*. **98**, 55–66 (1985), doi:10.1007/BF02488906.

40. A. Sasago, T. Sibaoka, Water extrusion in the Water extrusion in the trap bladders of *Utricularia vulgaris* II. A possible mechanism of water outflow. *The Botanical Magazine, Tokyo*. **98**, 113–124 (1985), doi:10.1007/BF02488791.

41. B. E. Juniper, R. J. Robins, D. M. Joel, *The carnivorous plants* (Academic Press, London, 1989).

42. L. Adamec, Functional characteristics of traps of aquatic carnivorous Utricularia species. *Aquatic Botany*. **95**, 226–233 (2011), doi:10.1016/j.aquabot.2011.07.001.

43. Y. Forterre, J. M. Skotheim, J. Dumais, L. Mahadevan, How the Venus flytrap snaps. *Nature*. **433**, 421–425 (2005), doi:10.1038/nature03185.

44. S. Poppinga, D. Correa, B. Bruchmann, A. Menges, T. Speck, Plant Movements as Concept Generators for the Development of Biomimetic Compliant Mechanisms. *Integrative and comparative biology*. **60**, 886–895 (2020), doi:10.1093/icb/icaa028.

45. F. E. Lloyd, Struktur und Funktion des Eintrittsmechanismus bei *Utricularia*. *Beihefte zum Botanischen Centralblatt A*. **54**, 292–320 (1936).
