# Supplementary figures and images for: Speaking valve with integrated biomimetic overpressure release and acoustic warning signal
Source: Sci Rep. 2024 Nov 4;14:26655. doi: 10.1038/s41598-024-77595-0 (PMC11535527; doi:10.1038/s41598-024-77595-0)

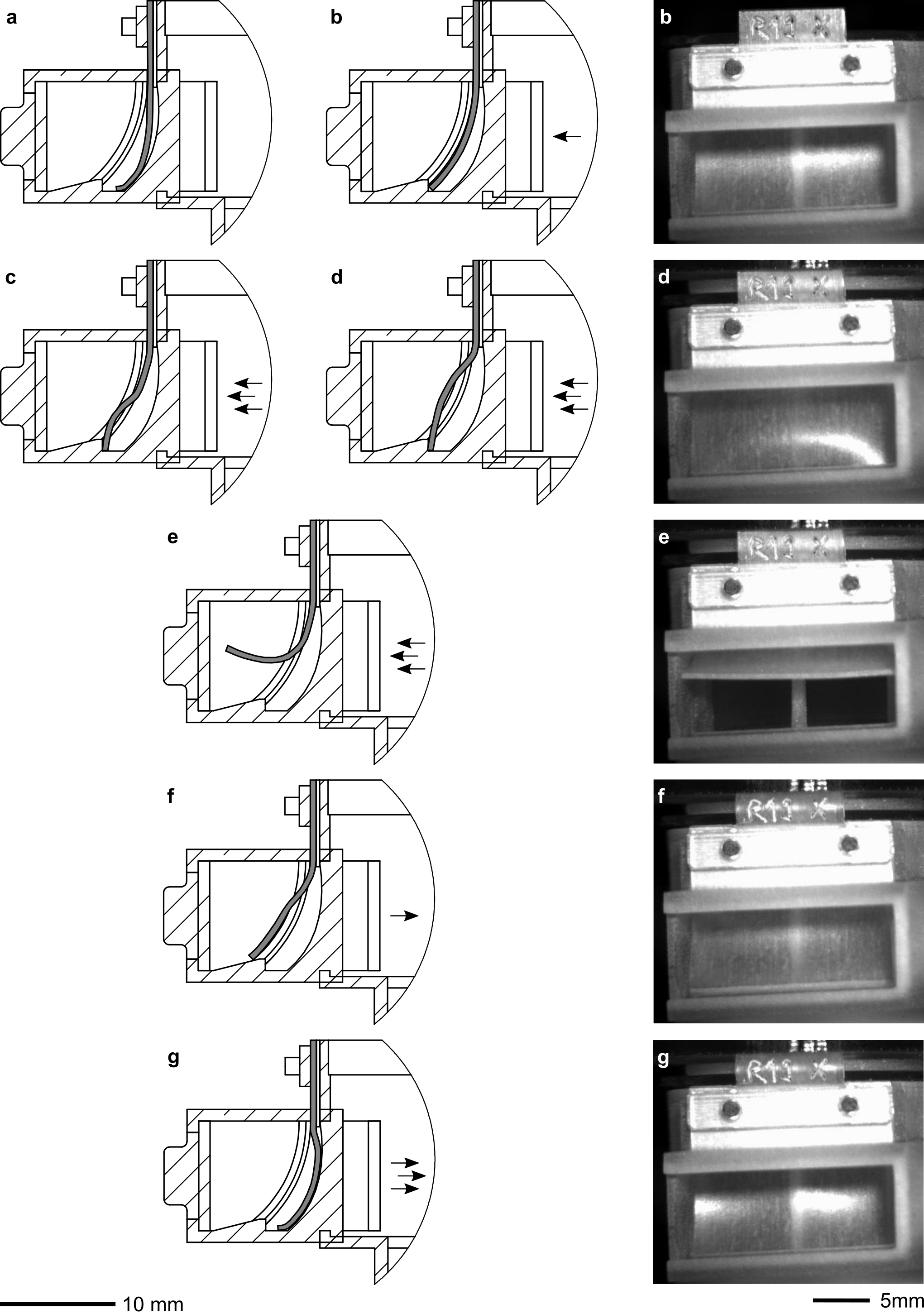

Supplement: Supplementary file 2 — Supplementary Material 2 [file 41598_2024_77595_MOESM2_ESM.jpg]

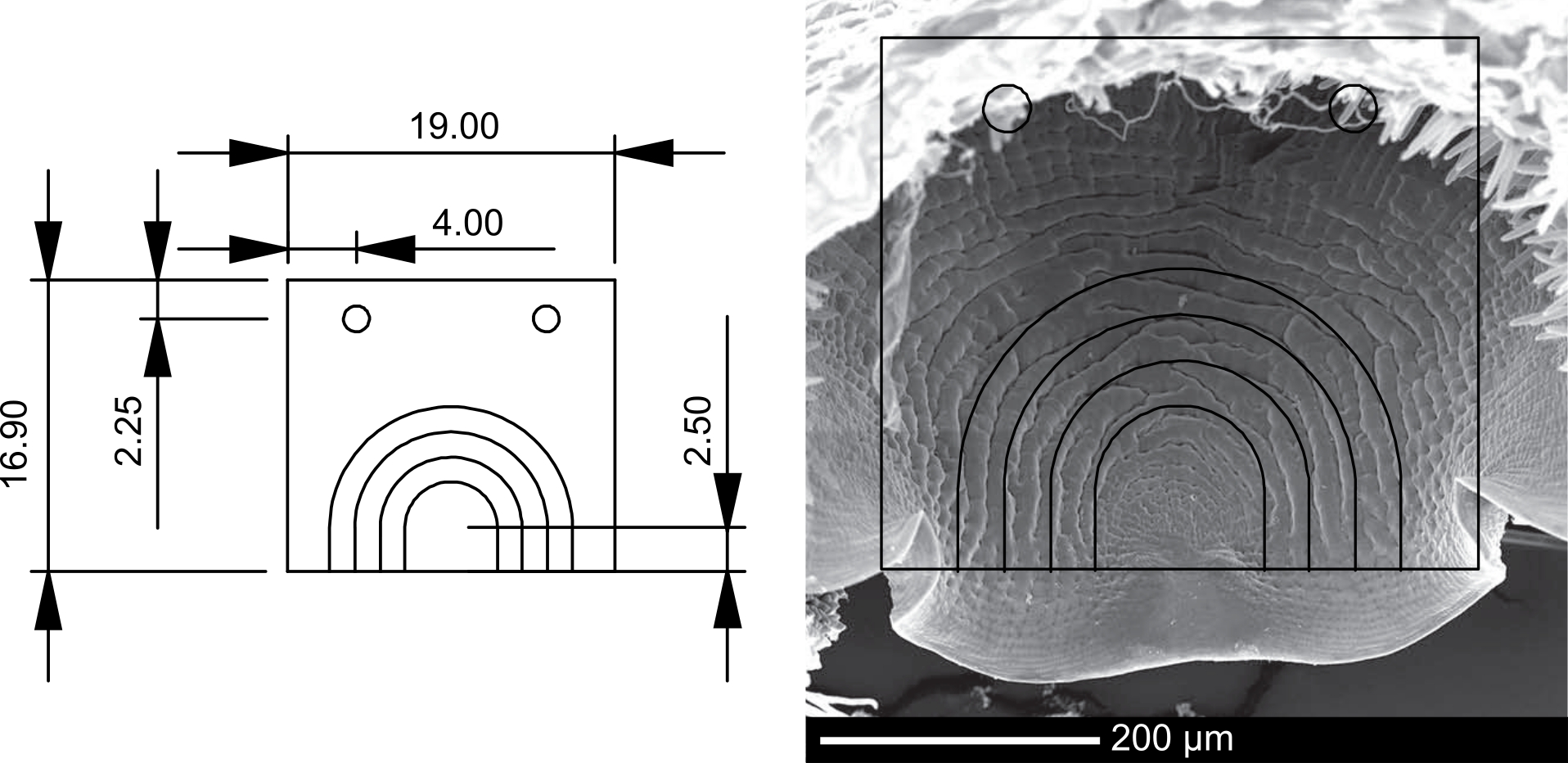

Supplement: Supplementary file 3 — Supplementary Material 3 [file 41598_2024_77595_MOESM3_ESM.jpg]

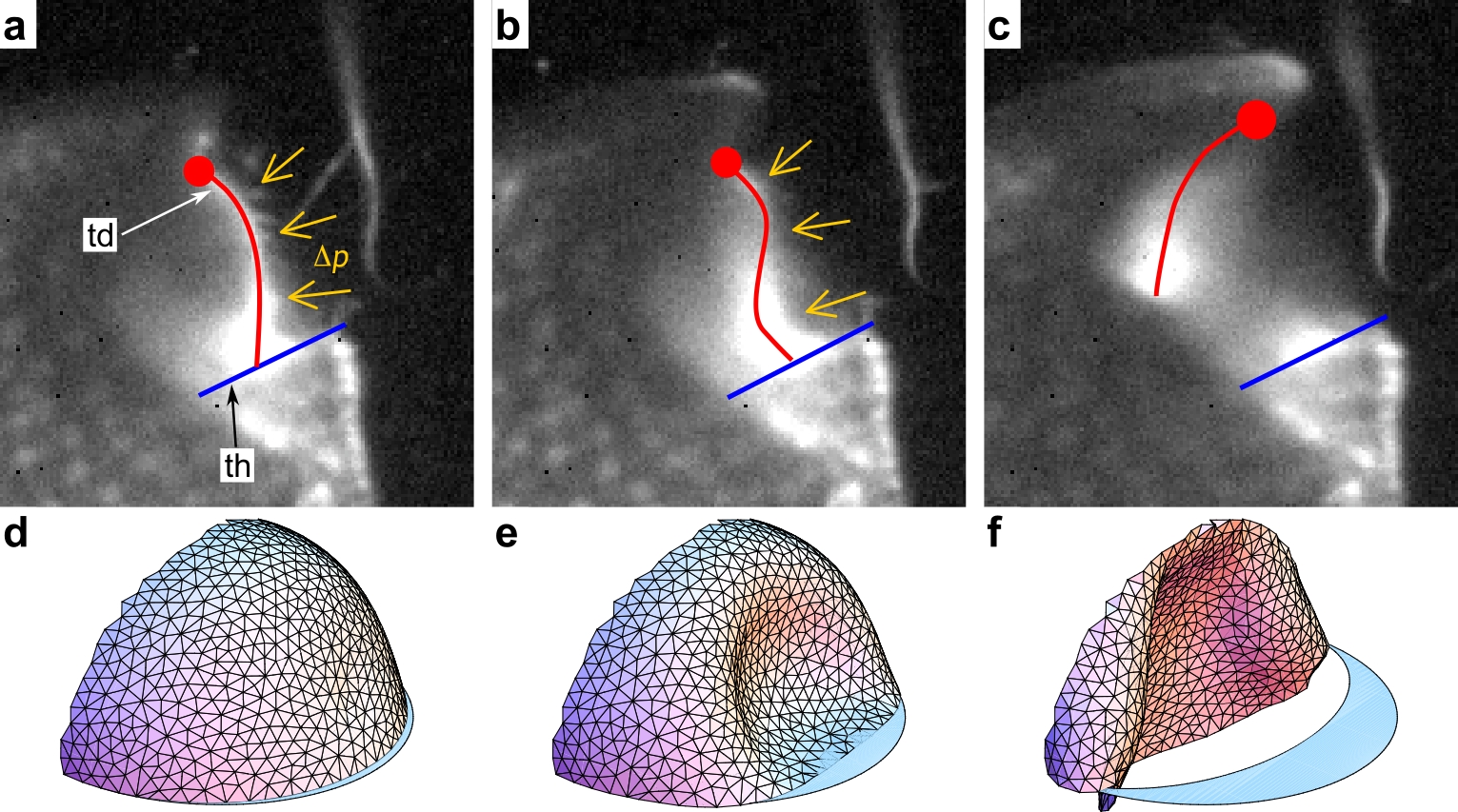

Supplement: Supplementary file 4 — Supplementary Material 4 [file 41598_2024_77595_MOESM4_ESM.jpg]

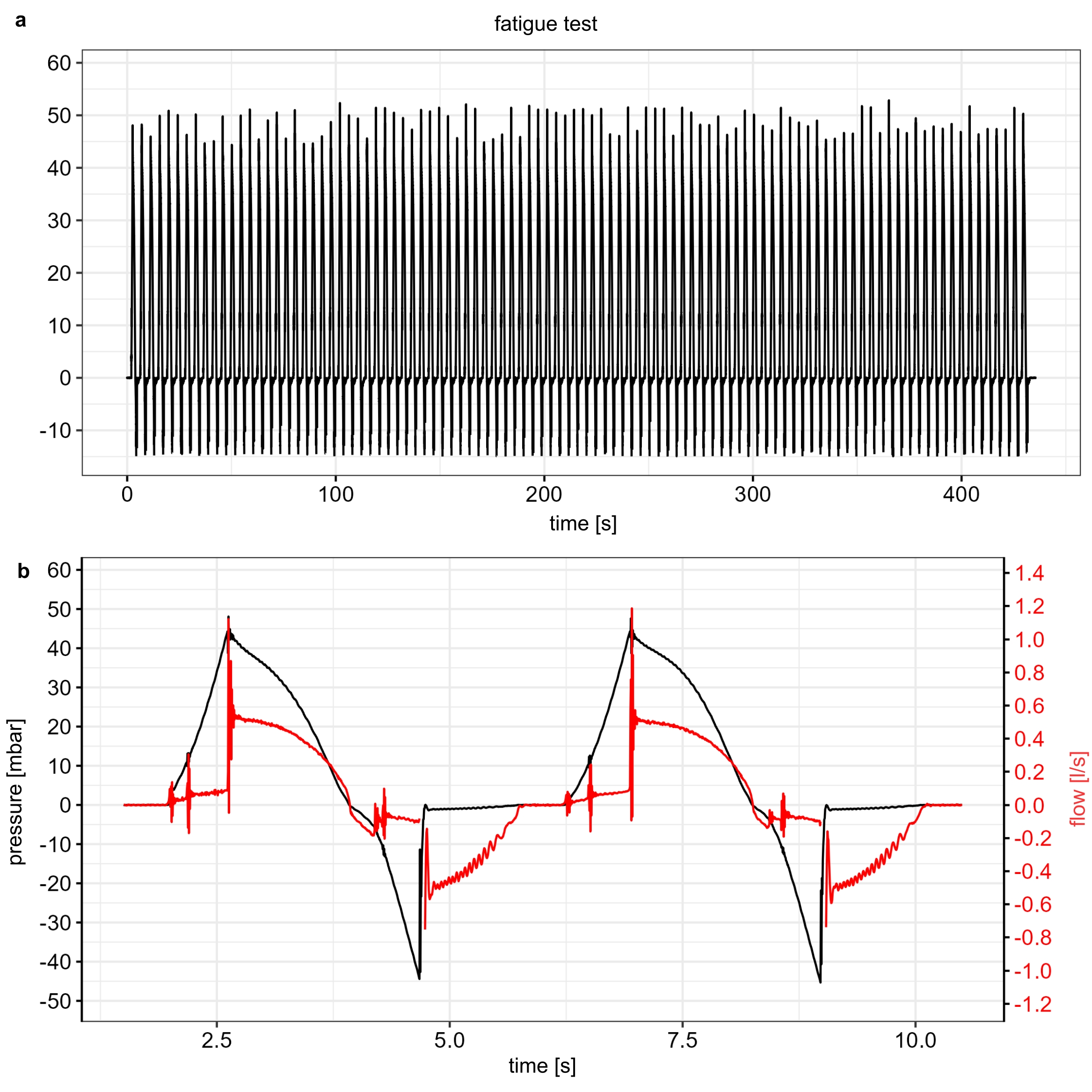

Supplement: Supplementary file 5 — Supplementary Material 5 [file 41598_2024_77595_MOESM5_ESM.jpg]

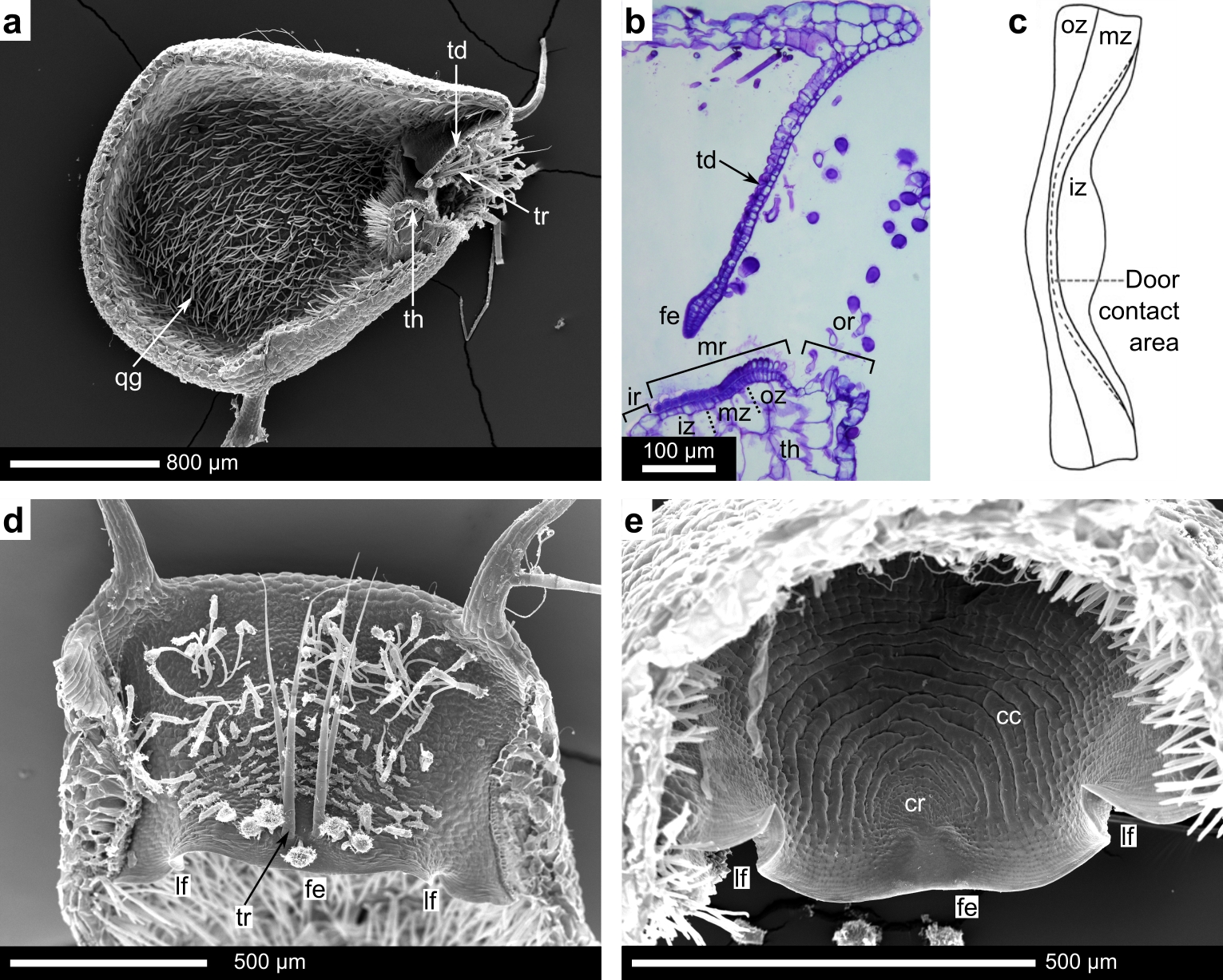

Supplement: Supplementary file 6 — Supplementary Material 6 [file 41598_2024_77595_MOESM6_ESM.jpg]

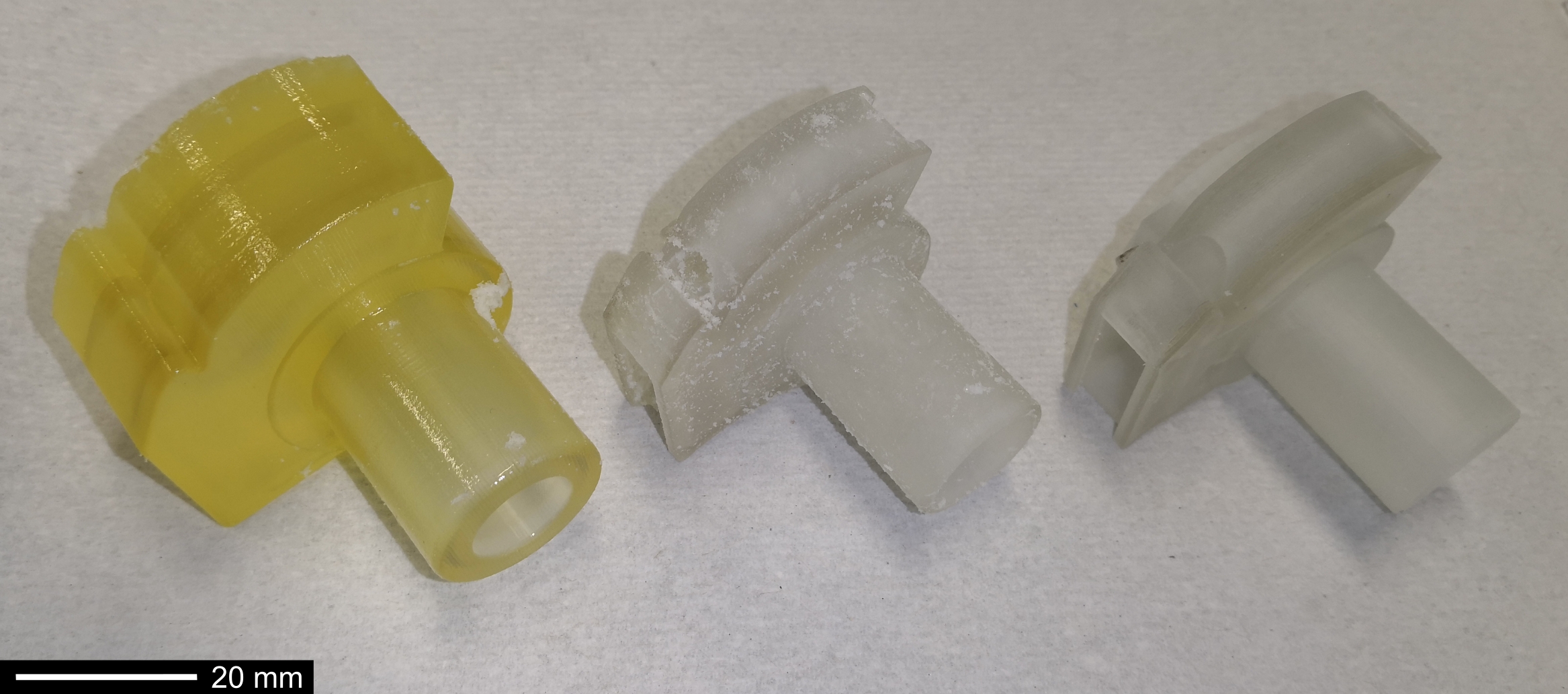

Supplement: Supplementary file 7 — Supplementary Material 7 [file 41598_2024_77595_MOESM7_ESM.jpg]
